# Timing of flowering and intensity of attack by a butterfly herbivore in a polyploid herb

**DOI:** 10.1002/ece3.1470

**Published:** 2015-04-12

**Authors:** Malin A E König, Christer Wiklund, Johan Ehrlén

**Affiliations:** 1 Department of Ecology, Environment and Plant Sciences, Stockholm UniversityStockholm, SE106 91, Sweden; 2 Department of Zoology, Stockholm UniversityStockholm, SE106 91, Sweden

**Keywords:** *Anthocharis cardamines*, *Cardamine pratensis*, cytotype, flowering phenology, herbivory, ontogeny

## Abstract

Timing of plant development both determines the abiotic conditions that the plant experiences and strongly influences the intensity of interactions with other organisms. Plants and herbivores differ in their response to environmental cues, and spatial and temporal variation in environmental conditions might influence the synchrony between host plants and herbivores, and the intensity of their interactions. We investigated whether differences in first day of flowering among and within 21 populations of the polyploid herb *Cardamine pratensis* influenced the frequency of oviposition by the butterfly *Anthocharis cardamines* during four study years. The proportion of plants that became oviposited upon differed among populations, but these differences were not related to mean flowering phenology within the population in any of the four study years. Attack rates in the field were also not correlated with resistance to oviposition estimated under controlled conditions. Within populations, the frequency of butterfly attack was higher in early-flowering individuals in two of the four study years, while there was no significant relationship in the other 2 years. Larger plants were more likely to become oviposited upon in all 4 years. The effects of first flowering day and size on the frequency of butterfly attack did not differ among populations. The results suggest that differences in attack intensities among populations are driven mainly by differences in the environmental context of populations while mean differences in plant traits play a minor role. The fact that within populations timing of flowering influenced the frequency of herbivore attack only in some years and suggests that herbivore-mediated selection on plant phenology differs among years, possibly because plants and herbivores respond differently to environmental cues.

## Introduction

A large fraction of selection experienced by organisms in ecological communities is the result of interactions with other species (Thompson 2014). In plants, spatial and temporal variation in interactions with herbivores and pollinators are important sources of variation in selection. For example, among-year variation in the relative timing of herbivores and their host plants might lead to variation both in the mean intensity of interactions and in selection on traits associated with plant developmental phenology (e.g., Hunter 1992). Many insect herbivores use cues associated with the floral display to locate their host plants, and in these systems, the overlap between the period of insect activity and host plant flowering strongly influences interaction strength and herbivore-mediated selection (Matter et al. 1999; Ehrlén 2014). Differences in the relative timing of plants and their herbivores among years can, in turn, be the result of differences in the response of developmental phenology to abiotic environmental cues, for example, temperature and changes in photoperiod (Visser and Both 2005; Forrest and Miller-Rushing 2010). Weather conditions, for example, in terms of temperature, wind, and precipitation, may also directly cause differences in the intensity of interactions among and within years (Peñuelas et al. 2002).

Environmental variation and differences in the response of interacting species to environmental cues might not only lead to temporal but also to spatial variation in interaction intensities and trait selection. Herbivores that can freely move among host plant populations in different environments should be able to exploit differences in phenology and preferentially use plant populations in suitable developmental phases (Hebblewhite et al. 2008). Such among-population differences in mean phenology could be the result of both genetic differentiation and habitat differences and should translate into differences in herbivore-mediated selection on plant flowering phenology (Pilson 2000; Post et al. 2008). Moreover, given that plants and herbivores use partly different environmental cues and that environmental conditions differ among years, we should expect herbivore-mediated selection on plant flowering phenology to differ between years (Mahoro 2002; Tarayre et al. 2007). Herbivore-mediated selection may vary also with the abundance of herbivores, and abundance differences may be the result of differences in habitat quality (Louda et al. 1990; Thompson 2005) and the abundance of natural enemies of herbivores (Bock et al. 1992). Lastly, the intensity of plant–herbivore interactions and herbivore-mediated selection on plant traits may vary among populations because of differences in plant resistance (Sturgeon 1979; Coley and Barone 1996). We still know little about how herbivore-mediated selection on plant flowering phenology varies among years and populations under natural conditions, and to what extent habitat differences versus variation in plant resistance contribute to differences among populations (but see Elle and Hare 2000; Valverde et al. 2001).

We investigated whether variation in flowering phenology, in terms of the date of the first open flower, of the polyploid herb *Cardamine pratensis* influenced the frequency of attack by the butterfly herbivore *Anthocharis cardamines*. We also examined whether these patterns differed among populations and years. As *A. cardamines* is a phenological specialist in terms of only ovipositing upon flowering plants, it is ideally suitable for investigating the effect of flowering phenology on oviposition patterns (Dempster 1997; Arvanitis et al. 2008). Moreover, herbivory by *A. cardamines* is usually associated with a large reduction in fitness for *C. pratensis*, because the growing larvae as a rule consume all seeds (Arvanitis et al. 2007). The leaves are also commonly damaged and the negative effects carry over to the next year (Boalt et al. 2010; König et al. 2014a). The ability to avoid oviposition by *A. cardamines* should thus be an important component of overall resistance to herbivory in *C. pratensis* in this region. For this study, 21 plant populations were followed during 4 years and information about flowering phenology and incidence of herbivore attack was collected from 1820 flowering events. These data were combined with results from a previous study (König et al. 2014b) that examined among-population variation in resistance, that is, the ability to avoid oviposition by *A. cardamines* under controlled conditions in *C. pratensis*, to disentangle effects of phenology versus effects of traits other than phenology on the frequency of butterfly oviposition. We used these data to ask two main questions: (1) Are differences in the mean intensity of attack by the butterfly *A. cardamines* among populations related to differences in mean flowering phenology and to differences in other traits associated with resistance? and (2) Is the incidence of butterfly attack within populations related to differences in flowering phenology among individuals and are these patterns consistent among years?

## Material and Methods

### Study system

*Cardamine pratensis* L. (Brassicaceae) is a common perennial polyploid herb in southern Sweden (Lövkvist 1956). Diploid populations are found in Europe but have never been found in Sweden (Lövkvist 1956). In our study area, situated within the Ludgo parish and within an 8.6 km radius from Tovetorps research station (south-central Sweden, 58.947086°N, 17.148854°E), tetraploids and octoploids are commonly found. Tetraploids mostly occur in open and dry meadows, while octoploids are typically found in damper and shadier habitats (Arvanitis et al. 2007). The two cytotypes are morphologically distinguishable; tetraploids are smaller and have more and smaller flowers than octoploids, and the leaf morphology differs between the two cytotypes (Lövkvist 1956). Both ploidy types flower from mid-May to mid-June in the south-central parts of Sweden, but tetraploid populations often start flowering 1–2 weeks earlier than octoploid populations (M. A. E. König pers. obs.), but this difference in phenology is not present in sympatric populations (Arvanitis et al. 2008). *Anthocharis cardamines* is the main herbivore on *C. pratensis* in south-central Sweden, but attacks by *Phyllotreta* sp. (Chrysomelidae, Coleoptera) and *Gastrophysa viridula* (Chrysomelidae, Coleoptera) larvae have been observed in some years (M. A. E. König pers. obs.).

The orange tip butterfly (*A. cardamines* L., Pieride) is obligately univoltine, overwinters in the pupal stage, and flies during May–June in Sweden. The butterfly uses several Brassicaceae species as hosts for its larvae, but often shows local specialization to one plant species (Wiklund 1984). *Cardamine pratensis* is one of the main butterfly host plants in our study area and tetraploid populations are often preferred over octoploid populations (Arvanitis et al. 2007). However, in sympatric populations, octoploid individuals are preferred over tetraploid individuals (Arvanitis et al. 2008). Differences in butterfly preferences for plant individuals within populations of the same ploidy type have been shown to be associated with differences in plant traits, such as the number of flowers, the width of the flower, and the size of the flower shoot (Arvanitis et al. 2008; König et al. 2014b). *Anthocharis cardamines* is a phenological specialist, that is, females only oviposit on flowering plants (Dempster 1997; Arvanitis et al. 2008) and avoid inflorescences older than 1 week (Dempster 1997). This avoidance might be due to a decrease of floral display size which might obstructs the ability of the butterfly female to locate the plant (Courtney 1982). The females are highly mobile and fly great distances in search for host plants, often passing through habitats unfit for oviposition (Wiklund and Åhrberg 1978; Arvanitis et al. 2007). Females recognize potential host plants at long distances by visual cues and are attracted to brightly colored objects within the size range of a Brassicaceae inflorescence (Wiklund and Åhrberg 1978). If the flowering stalk bends under the females weight when it lands on the inflorescence the female abandons the plant without oviposition. If the plant is considered suitable, the female bends its abdomen and deposits a single 1–2 mm large egg in the inflorescence, usually on the base of a flower or on the pedicel. The egg is deposited together with an oviposition deterrent pheromone that discourages other females from utilizing the same plant (Dempster 1992). The egg is white after oviposition, but turns orange during the following days, making detection relatively easy. The larva hatches within 7–10 days after oviposition and immediately begins to feed on the flower parts. As it grows, the larva will usually consume all fruits and most of the vegetative parts of the plant. When the fifth instar larval is fully grown, it enters a wandering phase and leaves the plant before choosing a suitable location for pupation.

### Study design

Within the study area, we followed 21 populations of *C. pratensis*, ten tetraploid, and eleven octoploid, during 2010–2013. Each year, up to 30 flowering individuals within each population were followed throughout the flowering season. In mid-May, before flowering had begun, individuals which were going to flower were located and marked by tying a small piece of yarn with a numbered piece of tape at the base of the flowering shoot. The flag was hidden under the vegetation between recordings to avoid influencing *A. cardamines* oviposition preferences. The number of flowering individuals within populations varied between 1 and 200 individuals while the number of nonflowering individuals was difficult to estimate because rosettes are inconspicuous.

Once a week during the flowering season (5–7 weeks), we searched for eggs and larvae of *A. cardamines*. The visits were continued up to the point when all buds of the marked individuals had opened in a population. In each individual, we also counted the number of flower buds, flowers, and fruits at each visit. Lastly, we measured the height and basal diameter of the flower shoot in all populations at the time when all marked individuals in all populations had started to flower. The height of the flowering shoot was measured from the rosette up to the first flower and the basal diameter of the flower shoot was measured immediately above the rosette.

Shoot volume was estimated using the formula for calculating the volume of a cylinder, where the height of the flower shoot represented the height of the cylinder and the basal diameter of the flower shoot represented the diameter of a cylinder. The number of flowers produced by an individual was estimated at each visit by the sum of all flower buds, open flowers and fruits. We used the highest estimate recorded over the flowering season as an estimate of the total potential flower production, thus accounting for potential losses of flowers due to herbivory during the observation period. Both the number of flowers and shoot volume were log-transformed to achieve normal distribution. The transformed shoot volume and transformed flower number were highly correlated within each ploidy type (*r*_tetraploids_ = 0.60, *r*_octoploids_ = 0.50). We therefore calculated the first principal component between the two variables and used it as an overall estimate of plant size in the analyses. Lastly, first day of flowering was defined as the number of days from May 1 until the day when the first flower opened in an individual. We were able to directly record the exact week during which each individual started to flower. To increase the resolution, we also estimated when within this weekly interval the first flower opened. This estimation was based on that flowers of *C. pratensis* open sequentially in an acropetal fashion, and that the individuals had a different number of flowers open when they were first recorded to flower. For each individual, we thus regressed the number of open flowers at each visit with at least one open flower and at least one bud on the number of days since May 1. The date of the first open flower in each individual was then estimated using the regression function of each individual to calculate the date at which flower number equaled 1.

Resistance traits are often defined as traits reducing fitness losses due to herbivory, either via increased plant defense or through escape from herbivores (Belsky et al. 1993). Differences in the frequency of butterfly attack may be related to several plant traits other than first day of flowering. To partly adjust for differences among populations in other traits in analyses of the effects of first flowering day on ovipostion, we therefore included an estimate of the population’s mean plant resistance derived in a previous study with the same study system (König et al. 2014b). The previous study investigating resistance against oviposition aimed to control for habitat differences among populations and individuals as well as the effects of flowering phenology. The reason for removing effects of phenological state in that study and assessing the effects of all traits other than phenology on resistance was that we know that phenology strongly affects the butterfly’s oviposition preferences, and that it is plastic, that is, highly dependent on environmental conditions. Thus, plants of similar phenological state were used and oviposition preference was estimated in an experiment carried out under environmentally controlled conditions. For that study, 2–4 plant individuals from each of the 21 populations studied under natural conditions were cloned, and groups of 16 mixed plant individuals, eight tetraploid and eight octoploid individuals, were constructed. The plant groups were presented to a single-mated female butterfly in an enclosed area and the time from the release of the female until oviposition was recorded for each plant. To compare results between females, time to oviposition was standardized within each trial by subtracting the mean and divide by the standard deviation. The mean standardized time for each population was used as an estimate of resistance against oviposition: plants with values below zero were more susceptible to oviposition than the average, while plants with values above zero were more resistant against oviposition (for details see König et al. 2014b).

### Statistical analyses

All statistical analyses were performed in R 2.15.3 (R Core Team 2013). First, we tested whether attack intensity varied among populations and years using generalized linear models. We used the frequency of butterfly attack as response variable (0 or 1) and population identity, year, and the interaction term as explanatory factors. Year had a significant effect on oviposition patterns (Deviance = 6.16, *P* = 0.013), and we therefore ran separate models for each year in all the following analyses. In some years less than 15 of all individuals within the population flowered (Appendix S1), and these populations by year combinations were excluded from the analysis.

To examine the effect of variation in phenology among populations on intensity of *A. cardamines* attack, we used models that in addition to population mean first flowering day, also included population mean plant size, population mean plant resistance, and ploidy type as predictors. Intensity of *A. cardamines* attack was estimated as the proportion of plants oviposited upon in the population.

To examine the effect of variation in phenology among individual plants within populations on the frequency of butterfly attack (0 or 1), we used generalized linear models with a binominal response. To examine the potential effect of ploidy type on the relationships between plant traits and the frequency of butterfly attack, we first ran generalized mixed effect models (lme4-package in R, Bates et al. 2011) with plant size, flowering phenology, ploidy type, and the interaction between ploidy type and the two plant traits as fixed factors and populations as a random factor. Second, we ran generalized linear models with a structure similar to the first set, but without ploidy type and with population identity instead as a fixed factor. Models with ploidy as fixed factor and population as random factor and models with population as fixed factor yielded similar results. In the results section below, we therefore only present the results for the latter models (the full models and the results of models with ploidy as fixed factor are given in Appendix S2). To ensure that we had at least some variation in oviposition among individuals within populations, populations which had less than five plants oviposited upon were excluded from the analyses of within-population variation (Appendix S2).

## Results

**Figure 1:**
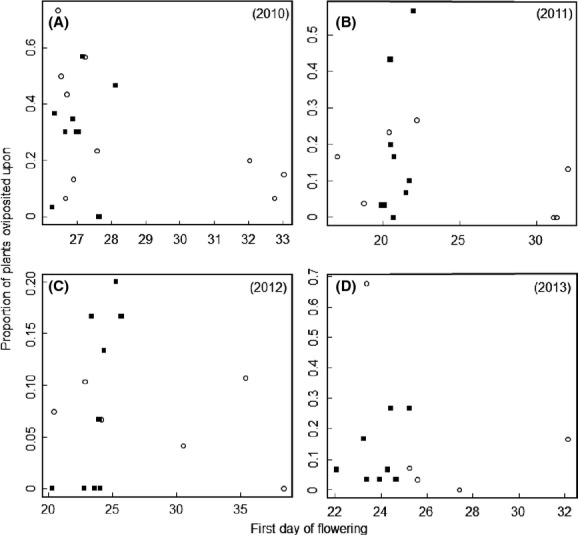
Relationship between first flowering day and the proportion of plant individuals in tetraploid (filled squares) and octoploid (open circles) populations of *Cardamine pratensis* oviposited upon by the butterfly herbivore *Anthocharis cardamines* in (A) 2010 (19 populations), (B) 2011 (16 populations), (C) 2012 (15 populations), and (D) 2013 (13 populations). None of the relationships are significant at *P* = 0.05.

**Figure 2:**
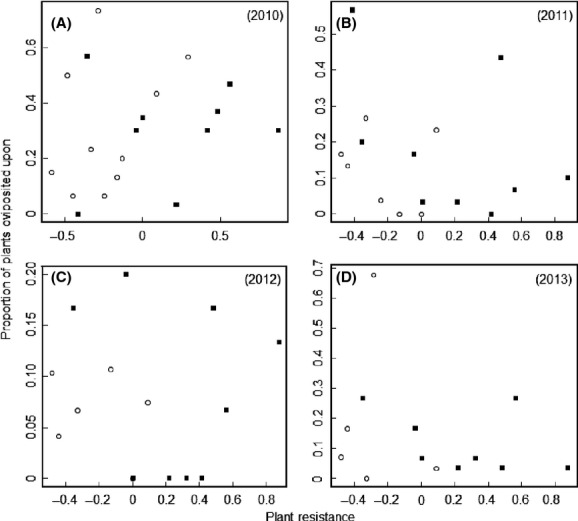
Relationship between plant resistance and the proportion of plant individuals in tetraploid (filled squares) and octoploid (open circles) populations of *Cardamine pratensis* oviposited upon by the butterfly herbivore *Anthocharis cardamines* in (A) 2010 (19 populations), (B) 2011 (16 populations), (C) 2012 (15 populations), and (D) 2013 (13 populations). Resistance was calculated by standardizing the time it took for a plant to receive an egg in a contextually controlled cage experiment by subtracting the mean oviposition time and dividing it by the standard deviation of the trial (for details see König et al. 2014a). Negative values of resistance represent low resistance against oviposition and positive values represent high resistance. None of the relationships are significant at *P* = 0.05.

In populations with butterfly oviposition, the proportion of flowering individuals that were attacked ranged from 3 to 73% during the four study years. The proportion of plants oviposited upon differed among both populations (Deviance = 62.26, *P* < 0.001) and years (Deviance = 6.16, *P* = 0.013, Appendix S1), but there was no significant effect of the interaction term (Deviance = 10.02, *P* = 0.19). The difference in butterfly attack rates among populations was not related to the mean date of first flowering in any of the 4 years (Table1, Fig.1). Plant size was significantly positively correlated with the proportion of plants being oviposited upon only in 2012 (Table1). Attack rates were not correlated with ploidy type or plant resistance against oviposition estimated under controlled conditions in any of the 4 years (Table1, Fig.2).

**Table 1:** The effect of population mean first flowering day, population mean resistance (standardized time until oviposition under controlled conditions), population mean plant size and ploidy, on the proportion of individuals in populations of *Cardamine pratensis* oviposited upon by the butterfly herbivore *Anthocharis cardamines* (2010: *n* = 19 populations; 2011: *n* = 16; 2012: *n* = 15; 2013: *n* = 13). Results are for linear models and for four flowering seasons. The effects of interactions between ploidy type and plant traits were never significant and are not presented

Year	Phenology				Resistance				Plant size					Ploidy type	
	*β*	df	*F*-value	*P*-value	*β*	df	*F*-value	*P*-value	*β*	df	*F*-value	*P*-value	df	*F*-value	*P*-value
2010	−0.03	1	1.51	0.24	0.09	1	0.27	0.61	0.10	1	0.09	0.70	1	0.70	0.42
2011	−0.01	1	0.16	0.70	−0.09	1	1.53	0.24	0.11	1	1.10	0.32	1	0.85	0.37
2012	0.00	1	0.17	0.69	−0.01	1	0.23	0.64	0.08	1	8.79	0.01	1	1.06	0.33
2013	−0.01	1	0.72	0.42	−0.12	1	0.21	0.66	0.08	1	0.15	0.71	1	0.29	0.61

**Figure 3:**
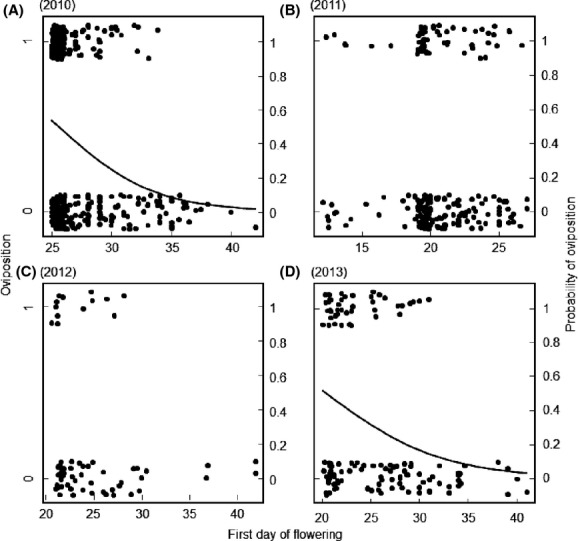
Relationship between first flowering day and the frequency of an individual plant of *Cardamine pratensis* being oviposited upon by the butterfly herbivore *Anthocharis cardamines* in (A) 2010 (plants: *n* = 361, populations: *n* = 13; *P* < 0.001), (B) 2011 (*n* = 186, *n* = 7, *P* = 0.88), (C) 2012 (*n* = 62, *n* = 3, *P* = 0.41) and (D) 2013 (*n* = 131, *n* = 5, *P* = 0.008). Each circle represents a plant individual. There was no significant effect of ploidy type and cytotypes are therefore not separated by different symbols in the figure (Appendix S2).

**Figure 4:**
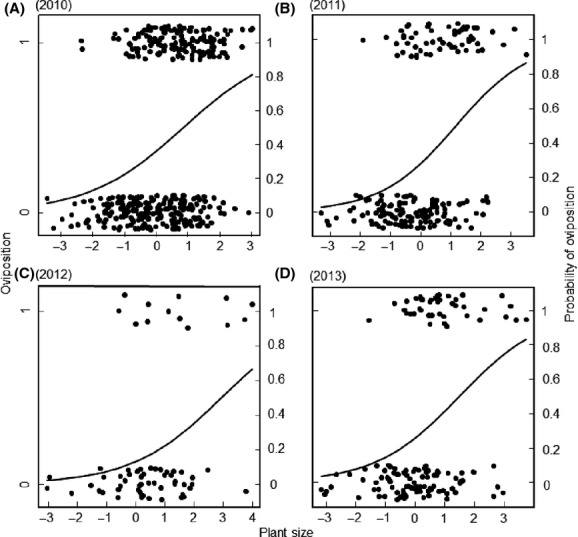
Relationship between plant size and the frequency of an individual plant of *Cardamine pratensis* being oviposited upon by the butterfly herbivore *Anthocharis cardamines* in (A) 2010 (plants: *n* = 361, populations: *n* = 13, *P* < 0.001), (B) 2011 (*n* = 186, *n* = 7, *P* < 0.001), (C) 2012 (*n* = 62, *n* = 3, *P* = 0.007), and (D) 2013 (*n* = 131, *n* = 5, *P* < 0.001). Each circle represents a plant individual. There was no significant effect of ploidy type and cytotypes are therefore not separated by different symbols in the figure (Appendix S2).

Within populations, individuals starting to flower earlier tended to be more likely to be attacked by the butterflies than later flowering individuals in all years, but the effect was only significant in 2010 and 2013 (the probability of attack decreased from 53% in the earliest flowering individual to 2% in the latest flowering in 2010, and from 52 to 2% in 2013, Table2, Fig.3). Larger plants were more likely to become oviposited upon in all years (the probability of attack increased from <10% in the smallest flowering plants to more than 60% in the largest plants in all 4 years, Fig.4). Population identity had a significant effect all years except in 2012 (Table2), but the interaction between population and first day of flowering was never significant (*P* > 0.05).

**Table 2:** The effect of first flowering day, plant size, and population identity on the frequency of an individual plant of *Cardamine pratensis* (2010: *n* = 361 individuals; 2011: *n* = 186; 2012: *n* = 62; 2013: *n* = 131) becoming oviposited upon by the butterfly herbivore *Anthocharis cardamines*. Results are for generalized linear models and for four flowering seasons

Years	Phenology					Plant size					Population		
	*β*_1_	SE	df	Deviance	*P*-value	*β*_1_	SE	df	Deviance	*P*-value	df	Deviance	*P*-value
2010	−0.16	0.06	1	14.56	<0.001	0.75	0.14	1	43.86	<0.001	12	35.60	<0.001
2011	−0.08	0.09	1	0.04	0.85	1.28	0.23	1	30.63	<0.001	6	40.84	<0.001
2012	−0.09	0.17	1	0.69	0.41	1.42	0.91	1	7.37	0.007	2	2.24	0.33
2013	−0.16	0.19	1	7.01	0.008	0.86	0.52	1	19.03	<0.001	4	26.75	<0.001

## Discussion

Our results with the butterfly *A. cardamines* and its host plant *C. pratensis* suggest that observed differences the intensity of the interaction among population were not related to differences in plant phenology and that associations between plant flowering phenology and frequency of herbivore attack preferences within populations varied among years. Within populations, early-flowering plants were significantly more likely to become oviposited upon than late flowering plants in two of four study years, while relationships were not significant in the other 2 years. A possible explanation for the observed differences in herbivore preferences among years is that the seasonal development of the herbivore and its host respond to partly different environmental cues, such as spring and winter temperatures, photoperiod, snowmelt, and soil moisture (van Asch and Visser 2007; Anderson et al. 2012; Ovaskainen et al. 2013), and that these cues vary in different ways among years.

A previous study in this system showed that *A. cardamines* preferred sunnier and more open habitats over shaded habitats (Arvanitis et al. 2007). The observed among-population and among-year variation in overall oviposition frequencies were thus expected due to habitat differences among plant populations and among-year variations in temperature. Similar spatial and temporal variations in herbivore frequencies have been observed in other systems and have been related to variation in several environmental factors (Chew and Courtney 1991; Huntly 1991; Cronin et al. 2001). Variation in mean first flowering day did not influence the proportion of plant individuals attacked within a population, meaning that the butterfly used populations independent on their phenological stage. Differences in mean plant size among populations had a significant effect only in 2012, when populations with larger individuals were more attacked than populations with smaller individuals. Growing larvae often consume all available plant material of *C. pratensis* before pupation (Arvanitis et al. 2008), but our results show that females do not always prefer the populations that contain individuals providing more food for the developing larvae. Lastly, ploidy type did not affect attack intensity, suggesting that genetic traits associated with polyploidization did not influence the females’ choice of populations. This result was in contrast to previous studies in this system that found higher frequencies of oviposition by *A. cardamines* in tetraploid populations (Arvanitis et al. 2007; Arvanitis et al. 2010).

In this study, we found no correlation between attack rates in the field and resistance estimated under controlled conditions. As resistance was estimated on plants grown under controlled conditions and all plants presented to the butterfly were in the same phenological stage (König et al. 2014b), this estimate includes the effects of all trait differences except phenology. The lack of correlation between resistance under controlled conditions and attack rates in the field thus suggests that the observed differences in attack rates among populations were due to environmental context (i.e., aspects of the environment influencing the herbivore directly, or indirectly, via plant phenotypic plasticity) rather than genetically based plant traits.

Within populations, the first day of flowering significantly influenced the frequency of individuals becoming oviposited upon in 2010 and 2013. In these years, earlier flowering individuals were more likely to become oviposited upon than later flowering ones. In 2011 and 2012, phenology did not significantly affect the frequency of butterfly attack. Still, the coefficient was negative in all 4 years, indicating that although the strength of the relationship varied among years the trend was always that plant individuals flowering early were more likely to become oviposited upon. One may hypothesize that if the sensitivity of the development rate to spring temperature differs between *A. cardamines* and *C. pratensis*, then the emergence of butterflies relative to their host plants should differ between years with different spring temperatures. This would in turn mean that butterflies should tend to oviposit mainly upon early-flowering individuals in some years, but use also later flowering plants in years when butterflies emerge later relative to the plant flowering phenology. However, testing this hypothesis requires additional experiments.

To conclude, our results suggest that selection among host plant populations is based mainly on habitat conditions, while plant traits, such as size and first flowering day, are important for selection of host plant individuals within populations, particularly in some years. The fact that we found no significant interactions between population identity and plant traits also indicates that butterfly females use the same cues within populations irrespective of the environmental setting. Plant flowering phenology influenced butterfly host plant selection only in some years, suggesting that climatic conditions and differences in the response to climate factors between the butterfly and the plant influence the synchrony between butterflies and host plants in this system. If plant and insect phenologies are differently affected by climatic factors, then strong relationships between plant phenology and intensities of interactions should only be detectable under some conditions, and the effect of variation in traits among and within populations should be difficult to evaluate under laboratory conditions or in single-year studies. To be able to fully understand how biotic agents such as pollinators and herbivores mediate selection on plant traits, such as flowering phenology, we have to examine relationships in natural populations during several years.

## Supporting Information

**Appendix S1.** Among year and population variation in number of plants oviposited upon in tetraploid and octoploid populations of *Cardamine pratensis*.

**Appendix S2.** Full models and results of the generalized mixed effect models in R 2.15.3 with ploidy type as fixed factor and population identity as random factor split per year.

**Appendix S3.** Data for the years 2010-2013 on population mean resistance under controlled conditions, population mean as well as data on individual plant traits for *Cardamine pratensis* and presence or absence of eggs by *Anthocharis cardamines*.
