# Supplementary material for: Timing of flowering and intensity of attack by a butterfly herbivore in a polyploid herb
Source: Ecol Evol. 2015 Apr 12;5(9):1863–72. doi: 10.1002/ece3.1470 (PMC4485967; doi:10.1002/ece3.1470)
Supplement: Supplementary file 1 [file ece30005-1863-sd1.doc]

Table S1. Characteristics of the 21 *Cardamine pratensis* populations included in the study: population name, ploidy type, population identity code, N and E coordinates of the populations, and the number of flowering individuals marked within each population in each of the study years.

| Pop. name | Ploidy type | Pop. code | Coordinates | Number of flowering individuals | | | |
| --- | --- | --- | --- | --- | --- | --- | --- |
|  |  |  |  | 2010 | 2011 | 2012 | 2013 |
| Bogslund | Tetraploids | 1 | 58.938918N, 17.081536E | 30 | 30 | 30 | 30 |
| Bysjön | Octoploids | 2 | 58.954186N, 17.108926E | 30 | 9 | 9 | 30 |
| Bölsäter ditch | Octoploids | 3 | 58.972969N, 17.146846E | 30 | 30 | 27 | 30 |
| Bölsäter meadow | Tetraploids | 4 | 58.972397N, 17.145285E | 26 | 30 | 30 | 30 |
| Dagnäs | Tetraploids | 5 | 59.009609N, 17.073144E | 30 | 30 | 14 | 3 |
| Dammen | Octoploids | 6 | 58.967423N, 17.080588E | 30 | 7 | 0 | 2 |
| Davik | Octoploids | 7 | 58.927006N, 17.119846E | 30 | 2 | 8 | 2 |
| Edeby | Tetraploids | 8 | 58.922106N, 17.044770E | 30 | 30 | 30 | 30 |
| Gustavsberg | Octoploids | 9 | 58.948443N, 17.069269E | 30 | 30 | 24 | 30 |
| Horssjön | Octoploids | 10 | 58.950938N, 17.035201E | 0 | 30 | 30 | 0 |
| Kallmyra 1 | Tetraploids | 11 | 58.881344N, 17.127420E | 30 | 30 | 30 | 30 |
| Kallmyra 3 | Tetraploids | 12 | 58.882606N, 17.123681E | 0 | 13 | 30 | 30 |
| Kryckeläng | Octoploids | 13 | 58.939502N, 17.113387E | 20 | 0 | 0 | 0 |
| Långbro | Octoploids | 14 | 58.945027N, 17.116214E | 30 | 26 | 1 | 2 |
| Norska hagen | Octoploids | 15 | 58.954665N, 17.094770E | 30 | 30 | 30 | 30 |
| Ryssinge 1 | Tetraploids | 16 | 58.929885N, 17.083156E | 30 | 30 | 30 | 30 |
| Ryssinge 2 | Tetraploids | 17 | 58.929509N, 17.088705E | 30 | 30 | 30 | 30 |
| Ryssinge 3 | Tetraploids | 18 | 58.931595N, 17.089282E | 30 | 30 | 30 | 30 |
| Svarvarn | Octoploids | 19 | 58.951200N, 17.073330E | 30 | 30 | 28 | 14 |
| Västra-malma meadow | Tetraploids | 20 | 58.956837N, 17.119926E | 30 | 30 | 30 | 30 |
| Västra-malma marsh | Octolploids | 21 | 58.958232N, 17.114685E | 30 | 30 | 29 | 28 |

Figure S1. Among-year and among-population variation in the proportion of plants oviposited upon in a) tetraploid and b) octoploid populations of *Cardamine pratensis* during 2010-2013*.* The numbers on the x-axis are the population codes (see Table S1).

Fig. 1.


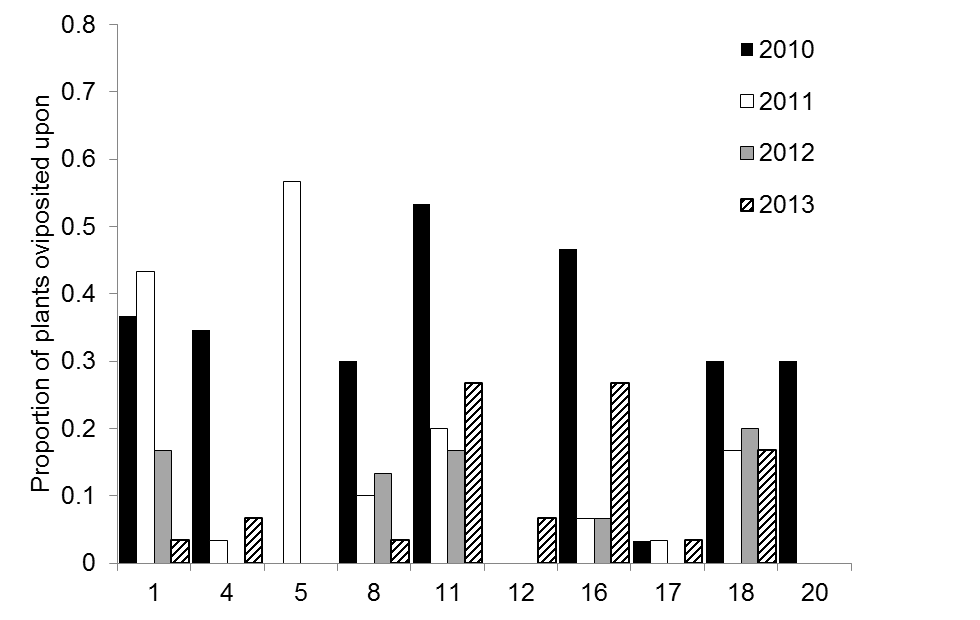


a


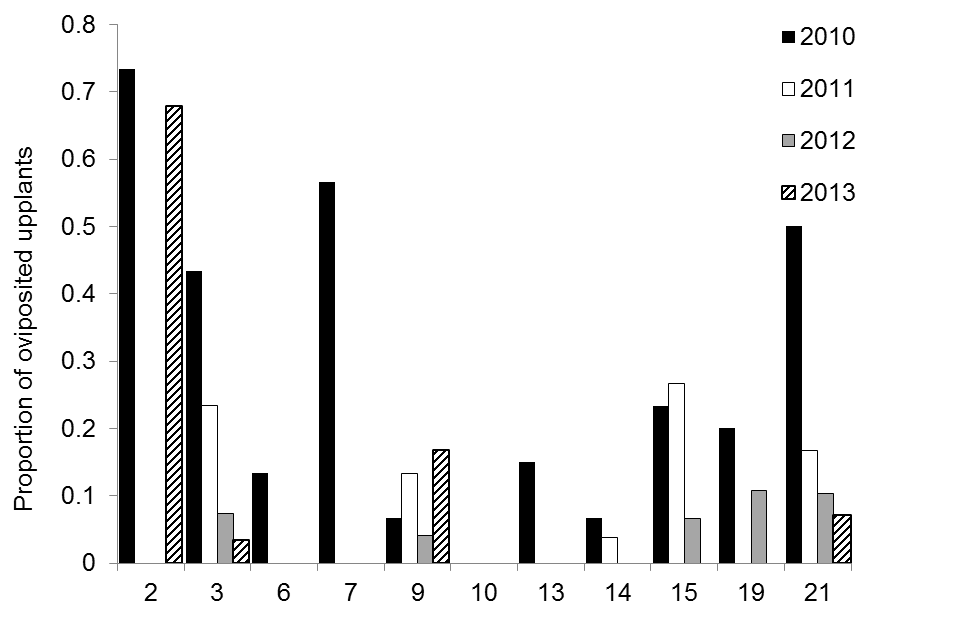


b
