# Supplementary material for: Timing of flowering and intensity of attack by a butterfly herbivore in a polyploid herb
Source: Ecol Evol. 2015 Apr 12;5(9):1863–72. doi: 10.1002/ece3.1470 (PMC4485967; doi:10.1002/ece3.1470)
Supplement: Supplementary file 2 [file ece30005-1863-sd2.doc]

**Appendix 2**

**Table A1.** Models of the likelihood of individuals of *Cardamine pratensis* to become oviposited by *Anthocharis cardamines* in 2010. The response (ovipositon) was binomial, a plant becoming oviposited (=1) or not (= 0). Ploidy type was used as a fixed factor and population identity (361 flowering individuals nested within 13 populations) as a random factor. Plant size is the first principle component of log transformed plant mass and log transformed number of flowers. Phenology is the number of days since first of May until first open flower. The results are presented in an ANOVA type III table, where the effect of the main effects is calculated without the non-significant interactions between plant traits and ploidy type. The model was analyzed using a generalized mixed effect model in the lme4 R-package.

Model with interaction term: glmer (Oviposition ~ (Plant size + Phenology) * Ploidy type + (1 | Population identity), family = binomial)

Model without interaction term: glmer (Oviposition ~ Plant size + Phenology + Ploidy type + (1 | Population identity), family = binomial)

| Random effects | Variance | Std. Dev. |
| --- | --- | --- |
| Plant population | 0.25 | 0.50 |

| Fixed effects – with interactions | z-value | P-value |
| --- | --- | --- |
| Plant size | 4.86 | <<0.001 |
| Phenology | -3.15 | 0.0016 |
| Ploidy type | -1.51 | 0.13 |
| Plant size : Ploidy type | 1.08 | 0.28 |
| Phenology : Ploidy type | 1.02 | 0.31 |

**Table A2.** Models of the likelihood of individuals of *C. pratensis* to become oviposited by *A. cardamines* in 2011. The response (ovipositon) was binomial, a plant becoming oviposited (=1) or not (= 0). Ploidy type was used as a fixed factor and population identity (186 flowering individuals nested within 7 populations) as a random factor. Plant size is the first principle component of log transformed plant mass and log transformed number of flowers. Phenology is the number of days since first of May until first open flower. The results are presented in an ANOVA type III table, where the effect of the main effects is calculated without the non-significant interactions between plant traits and ploidy type. The model was analyzed using a generalized mixed effect model in the lme4 R-package.

Model with interaction term: glmer (Oviposition ~ (Plant size + Phenology) * Ploidy type + (1 | Population identity), family = binomial)

Model without interaction term: glmer (Oviposition ~ Plant size + Phenology + Ploidy type + (1 | Population identity), family = binomial)

| Random effects | Variance | Std. Dev. |
| --- | --- | --- |
| Plant population | 0.96 | 1.00 |

| Fixed effects – with interactions | z-value | P-value |
| --- | --- | --- |
| Plant size | 5.37 | <<0.001 |
| Phenology | -0.72 | 0.47 |
| Ploidy type | 1.53 | 0.13 |
| Plant size : Ploidy type | 0.29 | 0.77 |
| Phenology : Ploidy type | 0.46 | 0.65 |

**Table A3.** Models of the likelihood of individuals of *C. pratensis* to become oviposited by *A.* *cardamines* in 2012. The response (ovipositon) was binomial, a plant becoming oviposited (=1) or not (= 0). 2012 only three tetraploid populations fulfilled the requirement of having at least 15 flowering individuals and at least five oviposited plants, ploidy type could thus not be used as a fixed factor this year. Population identity (62 flowering individuals nested within 3 populations) was used as a random factor. Plant size is the first principle component of log transformed plant mass and log transformed number of flowers. Phenology is the number of days since first of May until first open flower.The model was analyzed using a generalized mixed effect model in the lme4 R-package.

Model: glmer (Oviposition ~ Plant size + Phenology + (1 | Population identity), family = binomial)

| Random effects | Variance | Std. Dev. |
| --- | --- | --- |
| Plant population | 0.00 | 0.00 |

| Fixed effects – without interactions | z-value | P-value |
| --- | --- | --- |
| Plant size | 2.25 | 0.02 |
| Phenology | -0.79 | 0.43 |

**Table A4.** Models of the likelihood of individuals of *C. pratensis* to become oviposited by *A. cardamines* in 2013. The response (ovipositon) was binomial, a plant becoming oviposited (=1) or not (= 0). Ploidy type was used as a fixed factor and population identity (131 flowering individuals nested within 5 populations) as a random factor. Plant size is the first principle component of log transformed plant mass and log transformed number of flowers. Phenology is the number of days since first of May until first open flower. The results are presented in an ANOVA type III table, where the effect of the main effects is calculated without the non-significant interactions between plant traits and ploidy type. The model was analyzed using a generalized mixed effect model in the lme4 R-package.

Model with interaction term: glmer (Oviposition ~ (Plant size + Phenology) * Ploidy type + (1 | Population identity), family = binomial)

Model without interaction term: glmer (Oviposition ~ Plant size + Phenology + Ploidy type + (1 | Population identity), family = binomial)

| Random effects | Variance | Std. Dev. |
| --- | --- | --- |
| Plant population | 0.20 | 0.44 |

| Fixed effects – with interactions | z-value | P-value |
| --- | --- | --- |
| Plant size | 3.37 | 0.0003 |
| Phenology | -2.72 | 0.007 |
| Ploidy type | -2.28 | 0.02 |
| Plant size : Ploidy type | -0.47 | 0.64 |
| Phenology : Ploidy type | 1.42 | 0.16 |
