# Supplementary material for: Timing of flowering and intensity of attack by a butterfly herbivore in a polyploid herb
Source: Ecol Evol. 2015 Apr 12;5(9):1863–72. doi: 10.1002/ece3.1470 (PMC4485967; doi:10.1002/ece3.1470)
Supplement: Supplementary file 3 [file ece30005-1863-sd3.docx]

**Appendix 3.**

**Table A3-1. Data for the year 2010-2013 on population mean resistance under controlled conditions (Resist.), first day of flowering (FFF) and plants size of *Cardamine pratensis*, and the proportion of plants oviposited upon within the population (Attack) by *Anthocharis cardamines.* Populations with less than 15 flowering individuals were excluded from the analyses and data is not shown.**

|  |  |  | 2010 | | | 2011 | | | 2012 | | | 2013 | | |
| --- | --- | --- | --- | --- | --- | --- | --- | --- | --- | --- | --- | --- | --- | --- |
| Population identity | Ploidy | Resist. | Attack | FFF | Plant size | Attack | FFF | Plant size | Attack | FFF | Plant size | Attack | FFF | Plant size |
| Bogslund | Tetraploid | 0.48 | 0.37 | 26.36 | -0.65 | 0.43 | 20.52 | 0.15 | 0.17 | 25.72 | 0.24 | 0.03 | 24.65 | -0.11 |
| Bysjön | Octoploid | -0.29 | 0.73 | 26.44 | -0.72 |  |  |  |  |  |  | 0.68 | 23.36 | -0.39 |
| Bölsäter ditch | Octoploid | 0.09 | 0.43 | 26.71 | 0.09 | 0.23 | 20.42 | 0.47 | 0.07 | 20.44 | -0.74 | 0.03 | 25.59 | 0.12 |
| Bölsäter meadow | Tetraploid | 0.01 | 0.35 | 26.90 | 0.74 | 0.03 | 19.89 | 0.47 | 0.00 | 20.33 | 0.31 | 0.07 | 22.07 | 0.21 |
| Dagnäs | Tetraploid | -0.41 | 0.00 | 27.64 | 0.50 | 0.57 | 22.00 | 0.30 |  |  |  |  |  |  |
| Dammen | Octoploid | -0.16 | 0.13 | 26.91 | -0.94 |  |  |  |  |  |  |  |  |  |
| Davik | Octoploid | 0.29 | 0.57 | 27.24 | -0.16 |  |  |  |  |  |  |  |  |  |
| Edeby | Tetraploid | 0.88 | 0.30 | 26.97 | 0.53 | 0.10 | 21.75 | 0.43 | 0.13 | 24.35 | -0.65 | 0.03 | 23.95 | -0.07 |
| Gustavsberg | Octoploid | -0.44 | 0.07 | 32.75 | 0.31 | 0.13 | 31.99 | 0.79 | 0.04 | 30.54 | -0.21 | 0.17 | 32.14 | 0.09 |
| Horsjön | Octoploid |  |  |  |  | 0.00 | 31.07 | 1.11 | 0.00 | 38.36 | 0.55 |  |  |  |
| Kallmyra 1 | Tetraploid | -0.35 | 0.57 | 27.16 | 0.01 | 0.20 | 20.56 | 0.46 | 0.17 | 23.39 | -0.24 | 0.27 | 24.43 | -0.68 |
| Kallmyra 2 | Tetraploid |  |  |  |  |  |  |  | 0.00 | 23.66 | 0.47 | 0.07 | 24.29 | -0.30 |
| Kryckeläng | Octoploid | -0.58 | 0.15 | 33.02 | 0.97 |  |  |  |  |  |  |  |  |  |
| Långbro | Octoploid | -0.24 | 0.07 | 26.66 | 0.29 | 0.04 | 18.77 | 1.48 |  |  |  |  |  |  |
| Norska hagen | Octoploid | -0.33 | 0.23 | 27.58 | -0.25 | 0.27 | 22.18 | -0.89 | 0.07 | 24.18 | -0.33 | 0.00 | 27.42 | -0.93 |
| Ryssinge 1 | Tetraploid | 0.56 | 0.47 | 28.13 | -0.38 | 0.07 | 21.52 | 0.75 | 0.07 | 23.97 | 0.27 | 0.27 | 25.23 | 0.33 |
| Ryssinge 2 | Tetraploid | 0.22 | 0.03 | 26.28 | -0.35 | 0.03 | 20.14 | 0.61 | 0.00 | 22.81 | 0.12 | 0.03 | 23.39 | 0.25 |
| Ryssinge 3 | Tetraploid | -0.04 | 0.30 | 26.67 | -0.42 | 0.17 | 20.74 | -0.19 | 0.20 | 25.30 | -1.55 | 0.17 | 23.25 | -0.77 |
| Svarvarn | Octoploid | -0.13 | 0.20 | 32.04 | 0.01 | 0.00 | 31.29 | 0.08 | 0.11 | 35.42 | -0.73 |  |  |  |
| Västra-malma meadow | Tetraploid | 0.42 | 0.30 | 27.06 | -0.97 | 0.00 | 20.69 | 0.08 | 0.00 | 24.09 | 0.09 |  |  |  |
| Västra-malma marsh | Octoploid | -0.48 | 0.50 | 26.54 | 0.25 | 0.17 | 17.06 | 0.07 | 0.10 | 22.89 | 0.02 | 0.07 | 25.24 | 0.49 |

**Table A3-2.** Data for the years 2010-2013 on the first day of flowering, plant size and the presence or absence of *Anthocharis cardamines* egg on individual *Cardamine pratensis.* **Populations with less than 15 flowering individuals and less than five plants oviposited upon were excluded from the analyses and data is not shown.**

| Year | Population identity | Ploidy type | Oviposition | First flowering date | Plant size |
| --- | --- | --- | --- | --- | --- |
| 2010 | Bogslund | Tetraploid | 0 | 3.00 | 0.83 |
| 2010 | Bogslund | Tetraploid | 0 | 6.00 | -0.54 |
| 2010 | Bogslund | Tetraploid | 0 | 0.75 | -0.39 |
| 2010 | Bogslund | Tetraploid | 0 | 0.11 | 1.41 |
| 2010 | Bogslund | Tetraploid | 1 | 0.65 | 0.87 |
| 2010 | Bogslund | Tetraploid | 1 | 0.07 | 0.59 |
| 2010 | Bogslund | Tetraploid | 1 | 0.29 | -2.19 |
| 2010 | Bogslund | Tetraploid | 1 | 0.47 | -1.09 |
| 2010 | Bogslund | Tetraploid | 0 | 0.24 | -2.44 |
| 2010 | Bogslund | Tetraploid | 0 | 3.14 | -1.27 |
| 2010 | Bogslund | Tetraploid | 1 | 0.14 | -1.77 |
| 2010 | Bogslund | Tetraploid | 0 | 0.34 | -0.10 |
| 2010 | Bogslund | Tetraploid | 1 | 0.32 | -1.72 |
| 2010 | Bogslund | Tetraploid | 0 | 0.27 | -1.25 |
| 2010 | Bogslund | Tetraploid | 1 | 0.37 | -0.80 |
| 2010 | Bogslund | Tetraploid | 1 | 0.44 | -0.08 |
| 2010 | Bogslund | Tetraploid | 0 | 0.44 | -0.54 |
| 2010 | Bogslund | Tetraploid | 0 | 0.44 | -0.45 |
| 2010 | Bogslund | Tetraploid | 0 | 0.09 | -0.30 |
| 2010 | Bogslund | Tetraploid | 0 | 2.33 | -0.74 |
| 2010 | Bogslund | Tetraploid | 1 | 0.15 | -2.13 |
| 2010 | Bogslund | Tetraploid | 0 | 0.09 | -0.84 |
| 2010 | Bogslund | Tetraploid | 0 | 0.50 | -0.98 |
| 2010 | Bogslund | Tetraploid | 0 | 0.44 | -1.37 |
| 2010 | Bogslund | Tetraploid | 0 | 11.45 | 1.64 |
| 2010 | Bogslund | Tetraploid | 0 | 0.37 | -1.19 |
| 2010 | Bogslund | Tetraploid | 0 | 0.02 | -1.15 |
| 2010 | Bogslund | Tetraploid | 1 | 2.17 | -1.79 |
| 2010 | Bogslund | Tetraploid | 0 | 5.00 | 0.87 |
| 2010 | Bysjön | Octoploid | 1 | 0.44 | -2.20 |
| 2010 | Bysjön | Octoploid | 1 | 1.00 | 0.35 |
| 2010 | Bysjön | Octoploid | 0 | 1.24 | 0.77 |
| 2010 | Bysjön | Octoploid | 0 | 12.00 | 0.73 |
| 2010 | Bysjön | Octoploid | 1 | 3.75 | -0.15 |
| 2010 | Bysjön | Octoploid | 1 | 0.50 | -1.15 |
| 2010 | Bysjön | Octoploid | 1 | 0.87 | -0.07 |
| 2010 | Bysjön | Octoploid | 0 | 4.00 | 1.06 |
| 2010 | Bysjön | Octoploid | 1 | 0.13 | -1.95 |
| 2010 | Bysjön | Octoploid | 1 | 2.00 | 0.31 |
| 2010 | Bysjön | Octoploid | 0 | 1.00 | 0.28 |
| 2010 | Bysjön | Octoploid | 1 | 0.02 | -1.15 |
| 2010 | Bysjön | Octoploid | 0 | 2.20 | 0.05 |
| 2010 | Bysjön | Octoploid | 1 | 0.70 | -0.53 |
| 2010 | Bysjön | Octoploid | 0 | 0.78 | -1.32 |
| 2010 | Bysjön | Octoploid | 0 | 5.00 | 2.15 |
| 2010 | Bysjön | Octoploid | 1 | 0.33 | -2.30 |
| 2010 | Bysjön | Octoploid | 1 | 0.41 | -0.88 |
| 2010 | Bysjön | Octoploid | 1 | 0.08 | -1.10 |
| 2010 | Bysjön | Octoploid | 1 | 0.10 | -2.12 |
| 2010 | Bysjön | Octoploid | 1 | 0.70 | -1.10 |
| 2010 | Bysjön | Octoploid | 1 | 0.28 | -2.67 |
| 2010 | Bysjön | Octoploid | 1 | 0.32 | -1.88 |
| 2010 | Bysjön | Octoploid | 1 | 0.27 | -1.47 |
| 2010 | Bysjön | Octoploid | 1 | 0.41 | -2.11 |
| 2010 | Bysjön | Octoploid | 1 | 0.49 | -1.62 |
| 2010 | Bysjön | Octoploid | 1 | 0.15 | -0.78 |
| 2010 | Bysjön | Octoploid | 0 | 0.56 | 0.58 |
| 2010 | Bölsäter ditch | Octoploid | 0 | 3.10 | 1.15 |
| 2010 | Bölsäter ditch | Octoploid | 0 | 0.56 | -0.79 |
| 2010 | Bölsäter ditch | Octoploid | 1 | 8.08 | -1.67 |
| 2010 | Bölsäter ditch | Octoploid | 0 | 6.00 | 1.04 |
| 2010 | Bölsäter ditch | Octoploid | 0 | 5.00 | 1.54 |
| 2010 | Bölsäter ditch | Octoploid | 0 | 2.22 | 0.75 |
| 2010 | Bölsäter ditch | Octoploid | 1 | 0.48 | -1.43 |
| 2010 | Bölsäter ditch | Octoploid | 1 | 0.64 | -1.30 |
| 2010 | Bölsäter ditch | Octoploid | 1 | 0.13 | -0.71 |
| 2010 | Bölsäter ditch | Octoploid | 1 | 1.08 | 0.24 |
| 2010 | Bölsäter ditch | Octoploid | 0 | 1.67 | 1.82 |
| 2010 | Bölsäter ditch | Octoploid | 0 | 0.10 | -1.58 |
| 2010 | Bölsäter ditch | Octoploid | 1 | 0.90 | -1.11 |
| 2010 | Bölsäter ditch | Octoploid | 1 | 0.69 | -0.23 |
| 2010 | Bölsäter ditch | Octoploid | 0 | 0.63 | 0.51 |
| 2010 | Bölsäter ditch | Octoploid | 0 | 3.10 | 2.49 |
| 2010 | Bölsäter ditch | Octoploid | 1 | 4.00 | 0.97 |
| 2010 | Bölsäter ditch | Octoploid | 1 | 0.30 | 0.46 |
| 2010 | Bölsäter ditch | Octoploid | 0 | 0.94 | 0.31 |
| 2010 | Bölsäter ditch | Octoploid | 0 | 1.39 | -0.58 |
| 2010 | Bölsäter ditch | Octoploid | 1 | 0.51 | -0.44 |
| 2010 | Bölsäter ditch | Octoploid | 1 | 0.69 | -0.22 |
| 2010 | Bölsäter ditch | Octoploid | 0 | 2.22 | 1.50 |
| 2010 | Bölsäter ditch | Octoploid | 0 | 0.69 | -0.13 |
| 2010 | Bölsäter ditch | Octoploid | 0 | 1.09 | 0.36 |
| 2010 | Bölsäter ditch | Octoploid | 0 | 1.26 | 0.59 |
| 2010 | Bölsäter ditch | Octoploid | 1 | 0.90 | -0.51 |
| 2010 | Bölsäter ditch | Octoploid | 0 | 0.90 | -0.45 |
| 2010 | Bölsäter ditch | Octoploid | 0 | 0.90 | -0.09 |
| 2010 | Bölsäter meadow | Tetraploid | 1 | 0.76 | 2.39 |
| 2010 | Bölsäter meadow | Tetraploid | 0 | 0.75 | 1.88 |
| 2010 | Bölsäter meadow | Tetraploid | 0 | 3.00 | 2.49 |
| 2010 | Bölsäter meadow | Tetraploid | 0 | 0.21 | 1.10 |
| 2010 | Bölsäter meadow | Tetraploid | 0 | 1.56 | 0.69 |
| 2010 | Bölsäter meadow | Tetraploid | 1 | 0.97 | 0.19 |
| 2010 | Bölsäter meadow | Tetraploid | 0 | 4.50 | 1.84 |
| 2010 | Bölsäter meadow | Tetraploid | 0 | 0.10 | -1.16 |
| 2010 | Bölsäter meadow | Tetraploid | 0 | 0.27 | -0.25 |
| 2010 | Bölsäter meadow | Tetraploid | 1 | 0.64 | 0.97 |
| 2010 | Bölsäter meadow | Tetraploid | 0 | 0.48 | 0.61 |
| 2010 | Bölsäter meadow | Tetraploid | 0 | 0.30 | -0.01 |
| 2010 | Bölsäter meadow | Tetraploid | 0 | 0.49 | 0.94 |
| 2010 | Bölsäter meadow | Tetraploid | 1 | 0.22 | -0.62 |
| 2010 | Bölsäter meadow | Tetraploid | 1 | 0.82 | 0.61 |
| 2010 | Bölsäter meadow | Tetraploid | 0 | 0.69 | 1.06 |
| 2010 | Bölsäter meadow | Tetraploid | 0 | 0.00 | 0.50 |
| 2010 | Bölsäter meadow | Tetraploid | 1 | 0.98 | 1.33 |
| 2010 | Bölsäter meadow | Tetraploid | 1 | 0.23 | 0.21 |
| 2010 | Bölsäter meadow | Tetraploid | 1 | 0.38 | 0.78 |
| 2010 | Bölsäter meadow | Tetraploid | 0 | 0.26 | -0.06 |
| 2010 | Bölsäter meadow | Tetraploid | 0 | 0.85 | 2.01 |
| 2010 | Bölsäter meadow | Tetraploid | 0 | 10.99 | 1.52 |
| 2010 | Bölsäter meadow | Tetraploid | 0 | 9.19 | 0.56 |
| 2010 | Bölsäter meadow | Tetraploid | 1 | 8.87 | -1.09 |
| 2010 | Davik | Octoploid | 1 | 0.54 | 0.07 |
| 2010 | Davik | Octoploid | 0 | 8.00 | 2.59 |
| 2010 | Davik | Octoploid | 1 | 3.00 | 0.08 |
| 2010 | Davik | Octoploid | 1 | 0.90 | -0.15 |
| 2010 | Davik | Octoploid | 1 | 0.75 | -0.84 |
| 2010 | Davik | Octoploid | 1 | 0.32 | -1.35 |
| 2010 | Davik | Octoploid | 0 | 0.56 | -1.47 |
| 2010 | Davik | Octoploid | 0 | 0.55 | -2.10 |
| 2010 | Davik | Octoploid | 1 | 0.64 | -1.62 |
| 2010 | Davik | Octoploid | 1 | 0.69 | -1.03 |
| 2010 | Davik | Octoploid | 0 | 9.00 | 3.26 |
| 2010 | Davik | Octoploid | 0 | 4.00 | 0.41 |
| 2010 | Davik | Octoploid | 1 | 0.90 | -0.25 |
| 2010 | Davik | Octoploid | 0 | 0.95 | -0.88 |
| 2010 | Davik | Octoploid | 1 | 0.95 | -0.74 |
| 2010 | Davik | Octoploid | 1 | 1.11 | -0.29 |
| 2010 | Davik | Octoploid | 1 | 2.00 | 0.40 |
| 2010 | Davik | Octoploid | 1 | 0.90 | 0.41 |
| 2010 | Davik | Octoploid | 1 | 0.82 | -0.78 |
| 2010 | Davik | Octoploid | 0 | 0.49 | -0.56 |
| 2010 | Davik | Octoploid | 0 | 2.00 | 0.84 |
| 2010 | Davik | Octoploid | 0 | 4.00 | 0.76 |
| 2010 | Davik | Octoploid | 0 | 0.36 | -1.95 |
| 2010 | Davik | Octoploid | 1 | 1.70 | -0.10 |
| 2010 | Davik | Octoploid | 0 | 9.00 | 1.21 |
| 2010 | Edeby | Tetraploid | 1 | 0.09 | -2.98 |
| 2010 | Edeby | Tetraploid | 0 | 3.00 | 0.73 |
| 2010 | Edeby | Tetraploid | 1 | 0.02 | -0.62 |
| 2010 | Edeby | Tetraploid | 0 | 0.71 | -0.08 |
| 2010 | Edeby | Tetraploid | 0 | 1.29 | 2.69 |
| 2010 | Edeby | Tetraploid | 0 | 5.50 | 1.67 |
| 2010 | Edeby | Tetraploid | 0 | 0.89 | 0.35 |
| 2010 | Edeby | Tetraploid | 0 | 8.00 | 3.46 |
| 2010 | Edeby | Tetraploid | 0 | 0.91 | 0.28 |
| 2010 | Edeby | Tetraploid | 0 | 2.64 | -0.75 |
| 2010 | Edeby | Tetraploid | 0 | 0.64 | -0.60 |
| 2010 | Edeby | Tetraploid | 0 | 1.11 | 0.75 |
| 2010 | Edeby | Tetraploid | 0 | 4.00 | 2.99 |
| 2010 | Edeby | Tetraploid | 1 | 0.58 | -0.75 |
| 2010 | Edeby | Tetraploid | 1 | 0.50 | 0.22 |
| 2010 | Edeby | Tetraploid | 0 | 1.20 | 0.25 |
| 2010 | Edeby | Tetraploid | 0 | 5.34 | 0.63 |
| 2010 | Edeby | Tetraploid | 0 | 8.23 | 2.21 |
| 2010 | Edeby | Tetraploid | 0 | 0.04 | -1.29 |
| 2010 | Edeby | Tetraploid | 0 | 0.68 | 1.62 |
| 2010 | Edeby | Tetraploid | 0 | 0.50 | 1.09 |
| 2010 | Edeby | Tetraploid | 1 | 0.62 | 0.88 |
| 2010 | Edeby | Tetraploid | 1 | 0.77 | -0.84 |
| 2010 | Edeby | Tetraploid | 0 | 5.10 | 1.66 |
| 2010 | Edeby | Tetraploid | 1 | 0.67 | -0.64 |
| 2010 | Edeby | Tetraploid | 0 | 1.48 | 1.09 |
| 2010 | Edeby | Tetraploid | 1 | 2.00 | 0.60 |
| 2010 | Edeby | Tetraploid | 0 | 1.11 | 0.00 |
| 2010 | Edeby | Tetraploid | 0 | 0.90 | 0.65 |
| 2010 | Kallmyra 1 | Tetraploid | 1 | 0.33 | -1.02 |
| 2010 | Kallmyra 1 | Tetraploid | 1 | 5.33 | 0.63 |
| 2010 | Kallmyra 1 | Tetraploid | 1 | 0.53 | -0.22 |
| 2010 | Kallmyra 1 | Tetraploid | 1 | 0.73 | -0.04 |
| 2010 | Kallmyra 1 | Tetraploid | 0 | 12.43 | 1.77 |
| 2010 | Kallmyra 1 | Tetraploid | 1 | 0.32 | -0.16 |
| 2010 | Kallmyra 1 | Tetraploid | 1 | 0.07 | -1.48 |
| 2010 | Kallmyra 1 | Tetraploid | 1 | 0.13 | -0.77 |
| 2010 | Kallmyra 1 | Tetraploid | 0 | 0.29 | -1.09 |
| 2010 | Kallmyra 1 | Tetraploid | 1 | 0.89 | 0.38 |
| 2010 | Kallmyra 1 | Tetraploid | 1 | 0.07 | -0.24 |
| 2010 | Kallmyra 1 | Tetraploid | 1 | 0.14 | 0.18 |
| 2010 | Kallmyra 1 | Tetraploid | 0 | 17.00 | 0.28 |
| 2010 | Kallmyra 1 | Tetraploid | 0 | 1.18 | 0.53 |
| 2010 | Kallmyra 1 | Tetraploid | 0 | 1.72 | -0.52 |
| 2010 | Kallmyra 1 | Tetraploid | 0 | 2.00 | 1.36 |
| 2010 | Kallmyra 1 | Tetraploid | 1 | 0.19 | 0.24 |
| 2010 | Kallmyra 1 | Tetraploid | 0 | 0.51 | 0.66 |
| 2010 | Kallmyra 1 | Tetraploid | 0 | 0.16 | 1.30 |
| 2010 | Kallmyra 1 | Tetraploid | 1 | 1.57 | -0.48 |
| 2010 | Kallmyra 1 | Tetraploid | 1 | 6.86 | -0.67 |
| 2010 | Kallmyra 1 | Tetraploid | 0 | 0.14 | 0.43 |
| 2010 | Kallmyra 1 | Tetraploid | 0 | 3.00 | 1.12 |
| 2010 | Kallmyra 1 | Tetraploid | 0 | 0.55 | -0.40 |
| 2010 | Kallmyra 1 | Tetraploid | 0 | 0.67 | 0.94 |
| 2010 | Kallmyra 1 | Tetraploid | 1 | 0.59 | -1.96 |
| 2010 | Kallmyra 1 | Tetraploid | 1 | 0.50 | -0.68 |
| 2010 | Kallmyra 1 | Tetraploid | 0 | 0.24 | 0.29 |
| 2010 | Norska hagen | Octoploid | 0 | 0.60 | -1.71 |
| 2010 | Norska hagen | Octoploid | 1 | 0.75 | -1.63 |
| 2010 | Norska hagen | Octoploid | 0 | 1.94 | 0.77 |
| 2010 | Norska hagen | Octoploid | 1 | 3.05 | 0.60 |
| 2010 | Norska hagen | Octoploid | 0 | 1.00 | 0.01 |
| 2010 | Norska hagen | Octoploid | 1 | 2.00 | -0.53 |
| 2010 | Norska hagen | Octoploid | 1 | 1.00 | -0.45 |
| 2010 | Norska hagen | Octoploid | 0 | 9.00 | 0.18 |
| 2010 | Norska hagen | Octoploid | 0 | 3.00 | -0.66 |
| 2010 | Norska hagen | Octoploid | 0 | 3.00 | -0.82 |
| 2010 | Norska hagen | Octoploid | 0 | 4.00 | 0.47 |
| 2010 | Norska hagen | Octoploid | 0 | 0.75 | -0.77 |
| 2010 | Norska hagen | Octoploid | 0 | 5.00 | 0.25 |
| 2010 | Norska hagen | Octoploid | 0 | 1.00 | -1.49 |
| 2010 | Norska hagen | Octoploid | 0 | 1.00 | -0.32 |
| 2010 | Norska hagen | Octoploid | 0 | 1.80 | -0.68 |
| 2010 | Norska hagen | Octoploid | 0 | 0.47 | -2.00 |
| 2010 | Norska hagen | Octoploid | 0 | 2.78 | 0.22 |
| 2010 | Norska hagen | Octoploid | 0 | 5.00 | 0.56 |
| 2010 | Norska hagen | Octoploid | 0 | 9.00 | 0.92 |
| 2010 | Norska hagen | Octoploid | 0 | 9.00 | 2.24 |
| 2010 | Norska hagen | Octoploid | 0 | 3.05 | 1.53 |
| 2010 | Norska hagen | Octoploid | 0 | 1.00 | -0.73 |
| 2010 | Norska hagen | Octoploid | 1 | 0.82 | -1.36 |
| 2010 | Norska hagen | Octoploid | 0 | 0.05 | -1.32 |
| 2010 | Norska hagen | Octoploid | 0 | 0.01 | -1.24 |
| 2010 | Norska hagen | Octoploid | 1 | 5.00 | 0.84 |
| 2010 | Norska hagen | Octoploid | 0 | 0.94 | 0.84 |
| 2010 | Norska hagen | Octoploid | 1 | 0.56 | -1.06 |
| 2010 | Norska hagen | Octoploid | 0 | 0.79 | -0.20 |
| 2010 | Ryssinge 1 | Tetraploid | 0 | 10.00 | 2.31 |
| 2010 | Ryssinge 1 | Tetraploid | 1 | 0.28 | -0.22 |
| 2010 | Ryssinge 1 | Tetraploid | 0 | 11.00 | 2.18 |
| 2010 | Ryssinge 1 | Tetraploid | 1 | 0.64 | -0.80 |
| 2010 | Ryssinge 1 | Tetraploid | 0 | 0.42 | -0.90 |
| 2010 | Ryssinge 1 | Tetraploid | 0 | 0.39 | 0.12 |
| 2010 | Ryssinge 1 | Tetraploid | 0 | 11.21 | 0.01 |
| 2010 | Ryssinge 1 | Tetraploid | 1 | 0.22 | -1.44 |
| 2010 | Ryssinge 1 | Tetraploid | 0 | 1.04 | 1.23 |
| 2010 | Ryssinge 1 | Tetraploid | 0 | 1.28 | 1.17 |
| 2010 | Ryssinge 1 | Tetraploid | 0 | 9.51 | -1.32 |
| 2010 | Ryssinge 1 | Tetraploid | 1 | 0.73 | -1.77 |
| 2010 | Ryssinge 1 | Tetraploid | 1 | 0.50 | -0.57 |
| 2010 | Ryssinge 1 | Tetraploid | 0 | 0.33 | 0.01 |
| 2010 | Ryssinge 1 | Tetraploid | 0 | 4.33 | 0.59 |
| 2010 | Ryssinge 1 | Tetraploid | 0 | 6.33 | -0.56 |
| 2010 | Ryssinge 1 | Tetraploid | 1 | 0.40 | -2.09 |
| 2010 | Ryssinge 1 | Tetraploid | 0 | 0.40 | -1.48 |
| 2010 | Ryssinge 1 | Tetraploid | 1 | 1.00 | 0.70 |
| 2010 | Ryssinge 1 | Tetraploid | 0 | 0.69 | -1.03 |
| 2010 | Ryssinge 1 | Tetraploid | 1 | 1.47 | -0.35 |
| 2010 | Ryssinge 1 | Tetraploid | 1 | 0.76 | 0.20 |
| 2010 | Ryssinge 1 | Tetraploid | 0 | 3.12 | 1.66 |
| 2010 | Ryssinge 1 | Tetraploid | 0 | 4.98 | -1.01 |
| 2010 | Ryssinge 1 | Tetraploid | 0 | 9.00 | 0.13 |
| 2010 | Ryssinge 1 | Tetraploid | 1 | 0.02 | -0.73 |
| 2010 | Ryssinge 1 | Tetraploid | 1 | 3.79 | -2.66 |
| 2010 | Ryssinge 1 | Tetraploid | 1 | 7.14 | -1.64 |
| 2010 | Ryssinge 1 | Tetraploid | 1 | 2.33 | -1.77 |
| 2010 | Ryssinge 3 | Tetraploid | 1 | 0.53 | -1.37 |
| 2010 | Ryssinge 3 | Tetraploid | 0 | 0.50 | 0.16 |
| 2010 | Ryssinge 3 | Tetraploid | 0 | 0.54 | -1.79 |
| 2010 | Ryssinge 3 | Tetraploid | 0 | 0.63 | -0.67 |
| 2010 | Ryssinge 3 | Tetraploid | 0 | 1.00 | -0.93 |
| 2010 | Ryssinge 3 | Tetraploid | 1 | 0.20 | -1.50 |
| 2010 | Ryssinge 3 | Tetraploid | 1 | 1.04 | 0.27 |
| 2010 | Ryssinge 3 | Tetraploid | 1 | 0.40 | -0.35 |
| 2010 | Ryssinge 3 | Tetraploid | 1 | 2.00 | -1.59 |
| 2010 | Ryssinge 3 | Tetraploid | 0 | 0.22 | 1.25 |
| 2010 | Ryssinge 3 | Tetraploid | 0 | 0.22 | 0.91 |
| 2010 | Ryssinge 3 | Tetraploid | 1 | 0.58 | -1.54 |
| 2010 | Ryssinge 3 | Tetraploid | 1 | 0.75 | -0.05 |
| 2010 | Ryssinge 3 | Tetraploid | 0 | 10.66 | 0.50 |
| 2010 | Ryssinge 3 | Tetraploid | 1 | 0.33 | -1.64 |
| 2010 | Ryssinge 3 | Tetraploid | 0 | 0.18 | -1.63 |
| 2010 | Ryssinge 3 | Tetraploid | 0 | 0.15 | 0.98 |
| 2010 | Ryssinge 3 | Tetraploid | 0 | 0.60 | 0.28 |
| 2010 | Ryssinge 3 | Tetraploid | 0 | 0.33 | -1.32 |
| 2010 | Ryssinge 3 | Tetraploid | 0 | 0.03 | -1.43 |
| 2010 | Ryssinge 3 | Tetraploid | 0 | 3.31 | 0.78 |
| 2010 | Ryssinge 3 | Tetraploid | 0 | 0.76 | -0.48 |
| 2010 | Ryssinge 3 | Tetraploid | 0 | 7.00 | 1.42 |
| 2010 | Ryssinge 3 | Tetraploid | 0 | 0.40 | -0.22 |
| 2010 | Ryssinge 3 | Tetraploid | 1 | 0.22 | -1.25 |
| 2010 | Ryssinge 3 | Tetraploid | 0 | 0.34 | -0.81 |
| 2010 | Ryssinge 3 | Tetraploid | 1 | 5.43 | -0.53 |
| 2010 | Ryssinge 3 | Tetraploid | 0 | 0.16 | -2.07 |
| 2010 | Ryssinge 3 | Tetraploid | 0 | 4.00 | 0.74 |
| 2010 | Ryssinge 3 | Tetraploid | 0 | 0.02 | -0.75 |
| 2010 | Ryssinge 3 | Tetraploid | 0 | 7.99 | 0.79 |
| 2010 | Svarvarn | Octoploid | 0 | 10.00 | 2.70 |
| 2010 | Svarvarn | Octoploid | 0 | 11.26 | 0.31 |
| 2010 | Svarvarn | Octoploid | 1 | 5.80 | -0.86 |
| 2010 | Svarvarn | Octoploid | 0 | 7.20 | -0.41 |
| 2010 | Svarvarn | Octoploid | 0 | 14.96 | 1.63 |
| 2010 | Svarvarn | Octoploid | 1 | 7.20 | -0.02 |
| 2010 | Svarvarn | Octoploid | 0 | 8.75 | -0.67 |
| 2010 | Svarvarn | Octoploid | 0 | 3.00 | 0.32 |
| 2010 | Svarvarn | Octoploid | 0 | 1.53 | 0.20 |
| 2010 | Svarvarn | Octoploid | 0 | 6.50 | 0.45 |
| 2010 | Svarvarn | Octoploid | 0 | 7.67 | 1.10 |
| 2010 | Svarvarn | Octoploid | 0 | 7.25 | 0.39 |
| 2010 | Svarvarn | Octoploid | 1 | 0.62 | -1.33 |
| 2010 | Svarvarn | Octoploid | 0 | 7.25 | -0.41 |
| 2010 | Svarvarn | Octoploid | 0 | 6.51 | -1.35 |
| 2010 | Svarvarn | Octoploid | 0 | 5.76 | -1.08 |
| 2010 | Svarvarn | Octoploid | 0 | 9.00 | 0.68 |
| 2010 | Svarvarn | Octoploid | 0 | 7.67 | -0.58 |
| 2010 | Svarvarn | Octoploid | 1 | 4.98 | -0.24 |
| 2010 | Svarvarn | Octoploid | 0 | 10.62 | -0.08 |
| 2010 | Svarvarn | Octoploid | 0 | 3.00 | 0.28 |
| 2010 | Svarvarn | Octoploid | 1 | 1.19 | 0.60 |
| 2010 | Svarvarn | Octoploid | 1 | 4.40 | -0.90 |
| 2010 | Svarvarn | Octoploid | 0 | 7.25 | -0.42 |
| 2010 | Svarvarn | Octoploid | 0 | 6.00 | -1.23 |
| 2010 | Svarvarn | Octoploid | 0 | 10.00 | 1.13 |
| 2010 | Västra-malma marsh | Octoploid | 0 | 4.00 | 1.56 |
| 2010 | Västra-malma marsh | Octoploid | 1 | 0.64 | -0.24 |
| 2010 | Västra-malma marsh | Octoploid | 1 | 0.33 | -0.46 |
| 2010 | Västra-malma marsh | Octoploid | 0 | 3.00 | 2.08 |
| 2010 | Västra-malma marsh | Octoploid | 1 | 3.00 | 2.35 |
| 2010 | Västra-malma marsh | Octoploid | 1 | 0.30 | -0.65 |
| 2010 | Västra-malma marsh | Octoploid | 0 | 0.83 | 1.12 |
| 2010 | Västra-malma marsh | Octoploid | 0 | 4.00 | 1.42 |
| 2010 | Västra-malma marsh | Octoploid | 1 | 0.18 | 0.68 |
| 2010 | Västra-malma marsh | Octoploid | 1 | 4.00 | 1.37 |
| 2010 | Västra-malma marsh | Octoploid | 0 | 1.67 | -1.05 |
| 2010 | Västra-malma marsh | Octoploid | 1 | 2.56 | -1.76 |
| 2010 | Västra-malma marsh | Octoploid | 1 | 0.01 | -1.00 |
| 2010 | Västra-malma marsh | Octoploid | 1 | 0.01 | 0.07 |
| 2010 | Västra-malma marsh | Octoploid | 1 | 0.06 | -0.08 |
| 2010 | Västra-malma marsh | Octoploid | 0 | 0.01 | -0.27 |
| 2010 | Västra-malma marsh | Octoploid | 1 | 0.01 | -0.75 |
| 2010 | Västra-malma marsh | Octoploid | 0 | 0.78 | -0.87 |
| 2010 | Västra-malma marsh | Octoploid | 0 | 0.79 | -0.58 |
| 2010 | Västra-malma marsh | Octoploid | 1 | 3.00 | 1.22 |
| 2010 | Västra-malma marsh | Octoploid | 0 | 0.83 | 0.60 |
| 2010 | Västra-malma marsh | Octoploid | 1 | 1.45 | 0.76 |
| 2010 | Västra-malma marsh | Tetraploid | 1 | 0.02 | -2.94 |
| 2010 | Västra-malma meadow | Tetraploid | 1 | 0.74 | -0.58 |
| 2010 | Västra-malma meadow | Tetraploid | 0 | 0.45 | -0.55 |
| 2010 | Västra-malma meadow | Tetraploid | 0 | 1.05 | -0.49 |
| 2010 | Västra-malma meadow | Tetraploid | 0 | 0.03 | -0.18 |
| 2010 | Västra-malma meadow | Tetraploid | 0 | 0.26 | -0.82 |
| 2010 | Västra-malma meadow | Tetraploid | 0 | 0.60 | -0.86 |
| 2010 | Västra-malma meadow | Tetraploid | 0 | 2.00 | -0.53 |
| 2010 | Västra-malma meadow | Tetraploid | 0 | 0.02 | -0.60 |
| 2010 | Västra-malma meadow | Tetraploid | 1 | 2.48 | -1.23 |
| 2010 | Västra-malma meadow | Tetraploid | 0 | 2.42 | -1.12 |
| 2010 | Västra-malma meadow | Tetraploid | 1 | 2.64 | -1.45 |
| 2010 | Västra-malma meadow | Tetraploid | 0 | 5.56 | -0.45 |
| 2010 | Västra-malma meadow | Tetraploid | 1 | 3.92 | -2.04 |
| 2010 | Västra-malma meadow | Tetraploid | 0 | 0.47 | -1.65 |
| 2010 | Västra-malma meadow | Tetraploid | 0 | 0.36 | 0.28 |
| 2010 | Västra-malma meadow | Tetraploid | 0 | 0.29 | -0.48 |
| 2010 | Västra-malma meadow | Tetraploid | 0 | 4.08 | -0.68 |
| 2010 | Västra-malma meadow | Tetraploid | 0 | 6.47 | 0.35 |
| 2010 | Västra-malma meadow | Tetraploid | 0 | 13.00 | -0.75 |
| 2010 | Västra-malma meadow | Tetraploid | 0 | 1.00 | -1.59 |
| 2010 | Västra-malma meadow | Tetraploid | 1 | 0.14 | -1.45 |
| 2010 | Västra-malma meadow | Tetraploid | 1 | 0.47 | -1.72 |
| 2010 | Västra-malma meadow | Tetraploid | 0 | 0.09 | -0.72 |
| 2010 | Västra-malma meadow | Tetraploid | 0 | 5.40 | -0.44 |
| 2010 | Västra-malma meadow | Tetraploid | 0 | 0.24 | -0.85 |
| 2010 | Västra-malma meadow | Tetraploid | 1 | 3.48 | -1.72 |
| 2010 | Västra-malma meadow | Tetraploid | 0 | 3.00 | 0.45 |
| 2010 | Västra-malma meadow | Tetraploid | 1 | 0.85 | -1.43 |
| 2010 | Västra-malma meadow | Tetraploid | 0 | 0.25 | -2.87 |
| 2011 | Bogslund | Tetraploid | 0 | 13.66 | 0.64 |
| 2011 | Bogslund | Tetraploid | 1 | 7.43 | -1.88 |
| 2011 | Bogslund | Tetraploid | 1 | 7.49 | -1.56 |
| 2011 | Bogslund | Tetraploid | 1 | 7.09 | -0.65 |
| 2011 | Bogslund | Tetraploid | 0 | 7.13 | 0.33 |
| 2011 | Bogslund | Tetraploid | 0 | 7.36 | 0.32 |
| 2011 | Bogslund | Tetraploid | 0 | 7.54 | -0.85 |
| 2011 | Bogslund | Tetraploid | 1 | 9.16 | 1.13 |
| 2011 | Bogslund | Tetraploid | 1 | 7.74 | -2.76 |
| 2011 | Bogslund | Tetraploid | 1 | 9.20 | -1.89 |
| 2011 | Bogslund | Tetraploid | 0 | 11.24 | 0.29 |
| 2011 | Bogslund | Tetraploid | 0 | 7.85 | 1.32 |
| 2011 | Bogslund | Tetraploid | 0 | 10.08 | 1.92 |
| 2011 | Bogslund | Tetraploid | 1 | 7.62 | 1.92 |
| 2011 | Bogslund | Tetraploid | 0 | 8.43 | 2.34 |
| 2011 | Bogslund | Tetraploid | 0 | 8.75 | 1.28 |
| 2011 | Bogslund | Tetraploid | 1 | 7.50 | -0.32 |
| 2011 | Bogslund | Tetraploid | 1 | 7.43 | -1.51 |
| 2011 | Bogslund | Tetraploid | 0 | 8.98 | -0.80 |
| 2011 | Bogslund | Tetraploid | 0 | 14.52 | 2.00 |
| 2011 | Bogslund | Tetraploid | 0 | 7.51 | 0.02 |
| 2011 | Bogslund | Tetraploid | 0 | 6.90 | 0.70 |
| 2011 | Bogslund | Tetraploid | 0 | 11.74 | 1.48 |
| 2011 | Bogslund | Tetraploid | 1 | 7.43 | -1.38 |
| 2011 | Bogslund | Tetraploid | 0 | 7.15 | 0.51 |
| 2011 | Bogslund | Tetraploid | 0 | 6.86 | 1.02 |
| 2011 | Bogslund | Tetraploid | 1 | 8.63 | -0.28 |
| 2011 | Bogslund | Tetraploid | 0 | 7.67 | 0.50 |
| 2011 | Bogslund | Tetraploid | 0 | 7.75 | 0.47 |
| 2011 | Bölsäter ditch | Octoploid | 1 | 7.04 | -0.19 |
| 2011 | Bölsäter ditch | Octoploid | 1 | 12.18 | -1.24 |
| 2011 | Bölsäter ditch | Octoploid | 1 | 12.58 | 0.80 |
| 2011 | Bölsäter ditch | Octoploid | 1 | 7.42 | -1.28 |
| 2011 | Bölsäter ditch | Octoploid | 0 | 7.08 | -0.39 |
| 2011 | Bölsäter ditch | Octoploid | 0 | 8.00 | -0.18 |
| 2011 | Bölsäter ditch | Octoploid | 0 | 8.50 | 2.22 |
| 2011 | Bölsäter ditch | Octoploid | 0 | 7.70 | -0.14 |
| 2011 | Bölsäter ditch | Octoploid | 0 | 7.36 | -0.31 |
| 2011 | Bölsäter ditch | Octoploid | 0 | 7.82 | 0.61 |
| 2011 | Bölsäter ditch | Octoploid | 0 | 7.71 | 1.01 |
| 2011 | Bölsäter ditch | Octoploid | 1 | 7.71 | 0.65 |
| 2011 | Bölsäter ditch | Octoploid | 0 | 7.17 | -0.53 |
| 2011 | Bölsäter ditch | Octoploid | 0 | 7.43 | 1.25 |
| 2011 | Bölsäter ditch | Octoploid | 0 | 7.71 | 0.08 |
| 2011 | Bölsäter ditch | Octoploid | 0 | 12.00 | 3.05 |
| 2011 | Bölsäter ditch | Octoploid | 1 | 7.50 | -0.75 |
| 2011 | Bölsäter ditch | Octoploid | 0 | 9.67 | 0.70 |
| 2011 | Bölsäter ditch | Octoploid | 1 | 7.45 | -1.13 |
| 2011 | Bölsäter ditch | Octoploid | 0 | 8.58 | 1.60 |
| 2011 | Bölsäter ditch | Octoploid | 0 | 7.65 | 1.75 |
| 2011 | Bölsäter ditch | Octoploid | 0 | 7.27 | -0.90 |
| 2011 | Bölsäter ditch | Octoploid | 0 | 9.93 | 3.11 |
| 2011 | Bölsäter ditch | Octoploid | 0 | 7.55 | 1.29 |
| 2011 | Bölsäter ditch | Octoploid | 1 | 10.44 | -1.13 |
| 2011 | Bölsäter ditch | Octoploid | 0 | 7.36 | -0.08 |
| 2011 | Bölsäter ditch | Octoploid | 0 | 8.00 | 1.25 |
| 2011 | Bölsäter ditch | Octoploid | 0 | 10.25 | 1.22 |
| 2011 | Bölsäter ditch | Octoploid | 0 | 8.12 | 1.17 |
| 2011 | Dagnäs | Tetraploid | 0 | 7.85 | 1.27 |
| 2011 | Dagnäs | Tetraploid | 0 | 11.52 | 0.32 |
| 2011 | Dagnäs | Tetraploid | 0 | 13.00 | 0.77 |
| 2011 | Dagnäs | Tetraploid | 1 | 14.62 | 0.40 |
| 2011 | Dagnäs | Tetraploid | 1 | 7.86 | -0.24 |
| 2011 | Dagnäs | Tetraploid | 1 | 11.02 | -1.61 |
| 2011 | Dagnäs | Tetraploid | 1 | 12.65 | -1.23 |
| 2011 | Dagnäs | Tetraploid | 0 | 13.50 | -0.39 |
| 2011 | Dagnäs | Tetraploid | 1 | 9.62 | -0.19 |
| 2011 | Dagnäs | Tetraploid | 1 | 10.47 | 0.13 |
| 2011 | Dagnäs | Tetraploid | 1 | 7.67 | 0.16 |
| 2011 | Dagnäs | Tetraploid | 1 | 12.82 | 0.31 |
| 2011 | Dagnäs | Tetraploid | 0 | 8.11 | 1.31 |
| 2011 | Dagnäs | Tetraploid | 0 | 7.29 | 0.78 |
| 2011 | Dagnäs | Tetraploid | 1 | 11.00 | 1.91 |
| 2011 | Dagnäs | Tetraploid | 0 | 11.12 | 0.60 |
| 2011 | Dagnäs | Tetraploid | 1 | 7.42 | -0.37 |
| 2011 | Dagnäs | Tetraploid | 1 | 7.24 | 0.00 |
| 2011 | Dagnäs | Tetraploid | 1 | 7.13 | 0.18 |
| 2011 | Dagnäs | Tetraploid | 0 | 7.33 | 0.24 |
| 2011 | Dagnäs | Tetraploid | 0 | 9.00 | 1.39 |
| 2011 | Dagnäs | Tetraploid | 1 | 8.20 | 0.44 |
| 2011 | Dagnäs | Tetraploid | 1 | 11.62 | -1.67 |
| 2011 | Dagnäs | Tetraploid | 1 | 8.20 | 0.77 |
| 2011 | Dagnäs | Tetraploid | 1 | 13.69 | 0.59 |
| 2011 | Dagnäs | Tetraploid | 1 | 7.33 | -0.64 |
| 2011 | Dagnäs | Tetraploid | 0 | 15.00 | 2.79 |
| 2011 | Kallmyra 1 | Tetraploid | 0 | 7.35 | 1.72 |
| 2011 | Kallmyra 1 | Tetraploid | 1 | 8.59 | 0.14 |
| 2011 | Kallmyra 1 | Tetraploid | 1 | 1.00 | 0.29 |
| 2011 | Kallmyra 1 | Tetraploid | 0 | 13.00 | 2.13 |
| 2011 | Kallmyra 1 | Tetraploid | 0 | 6.88 | 0.41 |
| 2011 | Kallmyra 1 | Tetraploid | 1 | 7.35 | -1.77 |
| 2011 | Kallmyra 1 | Tetraploid | 0 | 7.80 | -0.41 |
| 2011 | Kallmyra 1 | Tetraploid | 0 | 6.14 | -0.30 |
| 2011 | Kallmyra 1 | Tetraploid | 0 | 9.00 | 1.32 |
| 2011 | Kallmyra 1 | Tetraploid | 0 | 9.29 | 1.39 |
| 2011 | Kallmyra 1 | Tetraploid | 0 | 7.67 | 1.25 |
| 2011 | Kallmyra 1 | Tetraploid | 0 | 10.76 | 0.99 |
| 2011 | Kallmyra 1 | Tetraploid | 0 | 9.00 | 1.63 |
| 2011 | Kallmyra 1 | Tetraploid | 0 | 7.55 | -0.32 |
| 2011 | Kallmyra 1 | Tetraploid | 1 | 7.32 | -1.37 |
| 2011 | Kallmyra 1 | Tetraploid | 0 | 7.67 | -0.31 |
| 2011 | Kallmyra 1 | Tetraploid | 0 | 7.67 | 0.95 |
| 2011 | Norska hagen | Octoploid | 0 | 7.49 | -0.45 |
| 2011 | Norska hagen | Octoploid | 0 | 10.65 | -2.07 |
| 2011 | Norska hagen | Octoploid | 0 | 7.04 | -0.54 |
| 2011 | Norska hagen | Octoploid | 1 | 7.51 | -0.94 |
| 2011 | Norska hagen | Octoploid | 1 | 9.25 | -1.50 |
| 2011 | Norska hagen | Octoploid | 0 | 7.20 | -2.07 |
| 2011 | Norska hagen | Octoploid | 0 | 10.48 | -0.95 |
| 2011 | Norska hagen | Octoploid | 0 | 12.61 | -1.41 |
| 2011 | Norska hagen | Octoploid | 0 | 9.07 | -0.51 |
| 2011 | Norska hagen | Octoploid | 0 | 14.02 | 0.15 |
| 2011 | Norska hagen | Octoploid | 0 | 7.58 | -0.54 |
| 2011 | Norska hagen | Octoploid | 1 | 7.51 | -1.91 |
| 2011 | Norska hagen | Octoploid | 0 | 8.62 | -1.26 |
| 2011 | Norska hagen | Octoploid | 0 | 8.48 | -0.94 |
| 2011 | Norska hagen | Octoploid | 0 | 13.63 | -0.06 |
| 2011 | Norska hagen | Octoploid | 0 | 15.00 | 0.89 |
| 2011 | Norska hagen | Octoploid | 0 | 10.48 | -0.98 |
| 2011 | Norska hagen | Octoploid | 1 | 11.24 | -3.12 |
| 2011 | Norska hagen | Octoploid | 0 | 13.00 | -0.15 |
| 2011 | Norska hagen | Octoploid | 0 | 12.00 | -0.44 |
| 2011 | Norska hagen | Octoploid | 0 | 11.13 | -0.42 |
| 2011 | Norska hagen | Octoploid | 0 | 8.78 | -0.44 |
| 2011 | Norska hagen | Octoploid | 1 | 7.18 | -1.03 |
| 2011 | Norska hagen | Octoploid | 0 | 11.65 | -1.29 |
| 2011 | Norska hagen | Octoploid | 1 | 11.75 | -1.13 |
| 2011 | Norska hagen | Octoploid | 0 | 13.83 | 0.46 |
| 2011 | Norska hagen | Octoploid | 0 | 9.69 | -1.15 |
| 2011 | Norska hagen | Octoploid | 1 | 13.71 | -0.42 |
| 2011 | Norska hagen | Octoploid | 1 | 8.51 | -1.73 |
| 2011 | Norska hagen | Octoploid | 0 | 6.33 | -0.59 |
| 2011 | Ryssinge 3 | Tetraploid | 1 | 7.13 | -2.05 |
| 2011 | Ryssinge 3 | Tetraploid | 0 | 9.00 | -1.68 |
| 2011 | Ryssinge 3 | Tetraploid | 0 | 8.53 | 1.80 |
| 2011 | Ryssinge 3 | Tetraploid | 0 | 14.00 | 0.62 |
| 2011 | Ryssinge 3 | Tetraploid | 0 | 7.35 | -0.74 |
| 2011 | Ryssinge 3 | Tetraploid | 0 | 13.33 | 0.82 |
| 2011 | Ryssinge 3 | Tetraploid | 0 | 7.64 | -0.76 |
| 2011 | Ryssinge 3 | Tetraploid | 0 | 7.78 | -0.04 |
| 2011 | Ryssinge 3 | Tetraploid | 0 | 7.42 | 1.21 |
| 2011 | Ryssinge 3 | Tetraploid | 0 | 13.00 | 3.29 |
| 2011 | Ryssinge 3 | Tetraploid | 0 | 7.58 | -0.94 |
| 2011 | Ryssinge 3 | Tetraploid | 0 | 7.54 | -1.54 |
| 2011 | Ryssinge 3 | Tetraploid | 1 | 7.58 | 0.56 |
| 2011 | Ryssinge 3 | Tetraploid | 0 | 10.99 | -0.13 |
| 2011 | Ryssinge 3 | Tetraploid | 0 | 7.36 | -1.51 |
| 2011 | Ryssinge 3 | Tetraploid | 1 | 12.19 | -1.60 |
| 2011 | Ryssinge 3 | Tetraploid | 0 | 7.22 | 0.82 |
| 2011 | Ryssinge 3 | Tetraploid | 0 | 7.20 | -0.09 |
| 2011 | Ryssinge 3 | Tetraploid | 0 | 7.78 | -0.54 |
| 2011 | Ryssinge 3 | Tetraploid | 0 | 11.48 | -2.19 |
| 2011 | Ryssinge 3 | Tetraploid | 0 | 9.33 | 0.09 |
| 2011 | Ryssinge 3 | Tetraploid | 0 | 8.00 | 1.46 |
| 2011 | Ryssinge 3 | Tetraploid | 0 | 9.04 | -0.36 |
| 2011 | Ryssinge 3 | Tetraploid | 1 | 7.33 | -0.95 |
| 2011 | Ryssinge 3 | Tetraploid | 0 | 7.03 | -0.25 |
| 2011 | Västra-malma marsh | Octoploid | 1 | 3.71 | -3.51 |
| 2011 | Västra-malma marsh | Octoploid | 0 | 2.21 | 0.00 |
| 2011 | Västra-malma marsh | Octoploid | 0 | 0.49 | -2.23 |
| 2011 | Västra-malma marsh | Octoploid | 0 | 4.60 | -1.85 |
| 2011 | Västra-malma marsh | Octoploid | 1 | 0.40 | -1.44 |
| 2011 | Västra-malma marsh | Octoploid | 1 | 5.10 | -2.40 |
| 2011 | Västra-malma marsh | Octoploid | 1 | 1.83 | 0.56 |
| 2011 | Västra-malma marsh | Octoploid | 1 | 1.78 | 0.43 |
| 2011 | Västra-malma marsh | Octoploid | 0 | 1.83 | 0.70 |
| 2011 | Västra-malma marsh | Octoploid | 0 | 0.81 | 0.84 |
| 2011 | Västra-malma marsh | Octoploid | 0 | 5.98 | 0.31 |
| 2011 | Västra-malma marsh | Octoploid | 0 | 3.33 | 0.66 |
| 2011 | Västra-malma marsh | Octoploid | 0 | 0.58 | 0.20 |
| 2011 | Västra-malma marsh | Octoploid | 0 | 1.14 | 1.00 |
| 2011 | Västra-malma marsh | Octoploid | 0 | 0.58 | 1.06 |
| 2011 | Västra-malma marsh | Octoploid | 0 | 1.00 | 1.11 |
| 2011 | Västra-malma marsh | Octoploid | 0 | 0.18 | 0.84 |
| 2011 | Västra-malma marsh | Octoploid | 0 | 4.26 | -0.13 |
| 2011 | Västra-malma marsh | Octoploid | 0 | 9.00 | 2.21 |
| 2011 | Västra-malma marsh | Octoploid | 0 | 8.00 | 0.19 |
| 2011 | Västra-malma marsh | Octoploid | 0 | 8.42 | 0.54 |
| 2011 | Västra-malma marsh | Octoploid | 0 | 13.00 | 1.42 |
| 2011 | Västra-malma marsh | Octoploid | 0 | 8.00 | 0.34 |
| 2011 | Västra-malma marsh | Octoploid | 0 | 8.42 | 0.59 |
| 2011 | Västra-malma marsh | Octoploid | 0 | 9.67 | 0.97 |
| 2011 | Västra-malma marsh | Octoploid | 0 | 11.00 | -0.17 |
| 2011 | Västra-malma marsh | Octoploid | 0 | 7.33 | -0.81 |
| 2011 | Västra-malma marsh | Octoploid | 0 | 10.53 | 0.63 |
| 2011 | Västra-malma marsh | Octoploid | 0 | 12.17 | 0.00 |
| 2012 | Bogslund | Tetraploid | 0 | 7.88 | 0.20 |
| 2012 | Bogslund | Tetraploid | 0 | 11.00 | 1.53 |
| 2012 | Bogslund | Tetraploid | 0 | 28.00 | 2.96 |
| 2012 | Bogslund | Tetraploid | 1 | 10.86 | -1.48 |
| 2012 | Bogslund | Tetraploid | 1 | 10.03 | -1.79 |
| 2012 | Bogslund | Tetraploid | 1 | 13.25 | 0.57 |
| 2012 | Bogslund | Tetraploid | 1 | 6.73 | -0.43 |
| 2012 | Bogslund | Tetraploid | 0 | 10.92 | 0.59 |
| 2012 | Bogslund | Tetraploid | 0 | 7.41 | -0.65 |
| 2012 | Bogslund | Tetraploid | 0 | 15.86 | -0.52 |
| 2012 | Bogslund | Tetraploid | 0 | 13.76 | -1.06 |
| 2012 | Bogslund | Tetraploid | 0 | 7.77 | -0.05 |
| 2012 | Bogslund | Tetraploid | 0 | 22.87 | -0.21 |
| 2012 | Bogslund | Tetraploid | 0 | 14.00 | 1.20 |
| 2012 | Bogslund | Tetraploid | 0 | 7.14 | -0.14 |
| 2012 | Bogslund | Tetraploid | 0 | 7.64 | 0.36 |
| 2012 | Bogslund | Tetraploid | 0 | 28.00 | 3.03 |
| 2012 | Kallmyra 1 | Tetraploid | 0 | 7.70 | -0.27 |
| 2012 | Kallmyra 1 | Tetraploid | 1 | 7.87 | -0.01 |
| 2012 | Kallmyra 1 | Tetraploid | 0 | 7.57 | -0.16 |
| 2012 | Kallmyra 1 | Tetraploid | 0 | 10.00 | 0.52 |
| 2012 | Kallmyra 1 | Tetraploid | 0 | 10.00 | 1.25 |
| 2012 | Kallmyra 1 | Tetraploid | 0 | 7.55 | -0.25 |
| 2012 | Kallmyra 1 | Tetraploid | 0 | 7.38 | -0.90 |
| 2012 | Kallmyra 1 | Tetraploid | 1 | 14.29 | 0.37 |
| 2012 | Kallmyra 1 | Tetraploid | 0 | 8.72 | -1.94 |
| 2012 | Kallmyra 1 | Tetraploid | 0 | 9.82 | -1.36 |
| 2012 | Kallmyra 1 | Tetraploid | 1 | 7.54 | -0.42 |
| 2012 | Kallmyra 1 | Tetraploid | 0 | 8.79 | -0.17 |
| 2012 | Kallmyra 1 | Tetraploid | 0 | 8.41 | -0.09 |
| 2012 | Kallmyra 1 | Tetraploid | 0 | 13.09 | -1.57 |
| 2012 | Kallmyra 1 | Tetraploid | 0 | 23.00 | 2.19 |
| 2012 | Kallmyra 1 | Tetraploid | 0 | 7.87 | -1.61 |
| 2012 | Kallmyra 1 | Tetraploid | 0 | 8.00 | -0.99 |
| 2012 | Kallmyra 1 | Tetraploid | 0 | 8.00 | 1.13 |
| 2012 | Kallmyra 1 | Tetraploid | 0 | 7.70 | -0.87 |
| 2012 | Kallmyra 1 | Tetraploid | 0 | 9.65 | 0.52 |
| 2012 | Kallmyra 1 | Tetraploid | 0 | 7.30 | -1.22 |
| 2012 | Kallmyra 1 | Tetraploid | 0 | 7.70 | 0.59 |
| 2012 | Ryssinge 3 | Tetraploid | 0 | 12.41 | -1.66 |
| 2012 | Ryssinge 3 | Tetraploid | 0 | 13.15 | -0.47 |
| 2012 | Ryssinge 3 | Tetraploid | 1 | 7.25 | -3.12 |
| 2012 | Ryssinge 3 | Tetraploid | 1 | 7.20 | -3.75 |
| 2012 | Ryssinge 3 | Tetraploid | 0 | 15.63 | -0.19 |
| 2012 | Ryssinge 3 | Tetraploid | 1 | 7.32 | -1.50 |
| 2012 | Ryssinge 3 | Tetraploid | 0 | 7.75 | -1.30 |
| 2012 | Ryssinge 3 | Tetraploid | 0 | 7.37 | -3.78 |
| 2012 | Ryssinge 3 | Tetraploid | 0 | 15.00 | -1.95 |
| 2012 | Ryssinge 3 | Tetraploid | 1 | 12.49 | -3.14 |
| 2012 | Ryssinge 3 | Tetraploid | 0 | 9.00 | -0.18 |
| 2012 | Ryssinge 3 | Tetraploid | 1 | 10.95 | -1.14 |
| 2012 | Ryssinge 3 | Tetraploid | 0 | 12.13 | -2.47 |
| 2012 | Ryssinge 3 | Tetraploid | 0 | 10.84 | -0.45 |
| 2012 | Ryssinge 3 | Tetraploid | 0 | 8.91 | -0.68 |
| 2012 | Ryssinge 3 | Tetraploid | 0 | 8.73 | -1.56 |
| 2012 | Ryssinge 3 | Tetraploid | 0 | 16.64 | 0.25 |
| 2012 | Ryssinge 3 | Tetraploid | 0 | 10.51 | -1.87 |
| 2012 | Ryssinge 3 | Tetraploid | 0 | 16.10 | -1.34 |
| 2012 | Ryssinge 3 | Tetraploid | 0 | 12.12 | -1.64 |
| 2012 | Ryssinge 3 | Tetraploid | 0 | 15.27 | -0.86 |
| 2012 | Ryssinge 3 | Tetraploid | 1 | 7.35 | -4.00 |
| 2012 | Ryssinge 3 | Tetraploid | 0 | 9.17 | 1.08 |
| 2013 | Bysjön | Octoploid | 1 | 25.00 | 1.55 |
| 2013 | Bysjön | Octoploid | 0 | 24.00 | 1.30 |
| 2013 | Bysjön | Octoploid | 0 | 20.87 | -0.04 |
| 2013 | Bysjön | Octoploid | 0 | 23.00 | 0.38 |
| 2013 | Bysjön | Octoploid | 0 | 25.83 | -0.45 |
| 2013 | Bysjön | Octoploid | 0 | 28.00 | 3.04 |
| 2013 | Bysjön | Octoploid | 0 | 27.00 | -0.71 |
| 2013 | Bysjön | Octoploid | 0 | 27.00 | -0.09 |
| 2013 | Bysjön | Octoploid | 1 | 20.35 | -2.34 |
| 2013 | Bysjön | Octoploid | 1 | 22.33 | -0.82 |
| 2013 | Bysjön | Octoploid | 1 | 23.00 | -1.36 |
| 2013 | Bysjön | Octoploid | 1 | 25.19 | -0.98 |
| 2013 | Bysjön | Octoploid | 1 | 25.38 | -2.18 |
| 2013 | Bysjön | Octoploid | 0 | 22.80 | 0.39 |
| 2013 | Bysjön | Octoploid | 1 | 20.70 | -0.80 |
| 2013 | Bysjön | Octoploid | 1 | 22.00 | -0.25 |
| 2013 | Bysjön | Octoploid | 1 | 20.87 | -0.47 |
| 2013 | Bysjön | Octoploid | 1 | 21.56 | -0.76 |
| 2013 | Bysjön | Octoploid | 1 | 27.78 | -0.20 |
| 2013 | Bysjön | Octoploid | 1 | 22.80 | -0.03 |
| 2013 | Bysjön | Octoploid | 1 | 25.25 | -1.07 |
| 2013 | Bysjön | Octoploid | 1 | 26.42 | -1.37 |
| 2013 | Bysjön | Octoploid | 1 | 20.29 | -1.25 |
| 2013 | Bysjön | Octoploid | 1 | 23.00 | -0.18 |
| 2013 | Bysjön | Octoploid | 1 | 20.58 | 0.36 |
| 2013 | Bysjön | Octoploid | 0 | 20.78 | -1.60 |
| 2013 | Bysjön | Octoploid | 1 | 21.30 | -0.69 |
| 2013 | Gustavsberg | Octoploid | 0 | 34.00 | -0.44 |
| 2013 | Gustavsberg | Octoploid | 1 | 22.00 | -1.75 |
| 2013 | Gustavsberg | Octoploid | 0 | 30.00 | 0.24 |
| 2013 | Gustavsberg | Octoploid | 1 | 30.00 | -0.21 |
| 2013 | Gustavsberg | Octoploid | 0 | 40.00 | 0.85 |
| 2013 | Gustavsberg | Octoploid | 0 | 38.00 | 0.19 |
| 2013 | Gustavsberg | Octoploid | 1 | 27.95 | -1.60 |
| 2013 | Gustavsberg | Octoploid | 1 | 29.00 | -0.57 |
| 2013 | Gustavsberg | Octoploid | 0 | 28.45 | -0.32 |
| 2013 | Gustavsberg | Octoploid | 0 | 31.00 | 0.31 |
| 2013 | Gustavsberg | Octoploid | 0 | 33.00 | 0.84 |
| 2013 | Gustavsberg | Octoploid | 0 | 31.00 | -0.49 |
| 2013 | Gustavsberg | Octoploid | 0 | 39.00 | 0.90 |
| 2013 | Gustavsberg | Octoploid | 0 | 39.00 | 0.41 |
| 2013 | Gustavsberg | Octoploid | 0 | 33.00 | -0.01 |
| 2013 | Gustavsberg | Octoploid | 1 | 29.30 | 0.28 |
| 2013 | Gustavsberg | Octoploid | 0 | 30.00 | -0.36 |
| 2013 | Gustavsberg | Octoploid | 0 | 30.32 | -1.25 |
| 2013 | Gustavsberg | Octoploid | 0 | 32.00 | 1.14 |
| 2013 | Gustavsberg | Octoploid | 0 | 33.00 | 0.72 |
| 2013 | Gustavsberg | Octoploid | 0 | 31.00 | 0.31 |
| 2013 | Gustavsberg | Octoploid | 0 | 34.00 | 1.22 |
| 2013 | Gustavsberg | Octoploid | 0 | 34.00 | 0.94 |
| 2013 | Gustavsberg | Octoploid | 0 | 31.33 | 0.03 |
| 2013 | Gustavsberg | Octoploid | 0 | 28.67 | -0.10 |
| 2013 | Gustavsberg | Octoploid | 0 | 34.00 | 0.62 |
| 2013 | Gustavsberg | Octoploid | 0 | 30.00 | 0.03 |
| 2013 | Gustavsberg | Octoploid | 0 | 28.20 | -1.07 |
| 2013 | Gustavsberg | Octoploid | 0 | 41.00 | 1.23 |
| 2013 | Gustavsberg | Octoploid | 0 | 32.00 | 0.60 |
| 2013 | Kallmyra 1 | Tetraploid | 1 | 20.83 | -0.50 |
| 2013 | Kallmyra 1 | Tetraploid | 0 | 21.19 | -0.17 |
| 2013 | Kallmyra 1 | Tetraploid | 1 | 20.91 | -1.11 |
| 2013 | Kallmyra 1 | Tetraploid | 1 | 23.16 | -0.49 |
| 2013 | Kallmyra 1 | Tetraploid | 1 | 21.67 | -0.54 |
| 2013 | Kallmyra 1 | Tetraploid | 0 | 20.77 | -1.96 |
| 2013 | Kallmyra 1 | Tetraploid | 0 | 21.21 | -0.42 |
| 2013 | Kallmyra 1 | Tetraploid | 1 | 21.34 | -1.64 |
| 2013 | Kallmyra 1 | Tetraploid | 0 | 21.58 | -0.78 |
| 2013 | Kallmyra 1 | Tetraploid | 1 | 20.65 | 0.29 |
| 2013 | Kallmyra 1 | Tetraploid | 0 | 20.70 | 0.60 |
| 2013 | Kallmyra 1 | Tetraploid | 0 | 20.20 | -0.37 |
| 2013 | Kallmyra 1 | Tetraploid | 0 | 27.14 | 0.37 |
| 2013 | Kallmyra 1 | Tetraploid | 0 | 20.72 | 0.28 |
| 2013 | Kallmyra 1 | Tetraploid | 0 | 23.55 | -0.28 |
| 2013 | Kallmyra 1 | Tetraploid | 0 | 34.65 | -0.53 |
| 2013 | Kallmyra 1 | Tetraploid | 0 | 34.16 | -0.94 |
| 2013 | Kallmyra 1 | Tetraploid | 1 | 22.49 | -3.40 |
| 2013 | Kallmyra 1 | Tetraploid | 0 | 34.24 | -1.38 |
| 2013 | Kallmyra 1 | Tetraploid | 0 | 20.71 | -0.63 |
| 2013 | Ryssinge 1 | Tetraploid | 0 | 25.24 | 1.03 |
| 2013 | Ryssinge 1 | Tetraploid | 0 | 28.44 | -0.17 |
| 2013 | Ryssinge 1 | Tetraploid | 0 | 28.00 | 1.39 |
| 2013 | Ryssinge 1 | Tetraploid | 0 | 21.28 | -1.36 |
| 2013 | Ryssinge 1 | Tetraploid | 0 | 21.25 | -0.24 |
| 2013 | Ryssinge 1 | Tetraploid | 0 | 28.87 | -0.05 |
| 2013 | Ryssinge 1 | Tetraploid | 0 | 21.64 | 1.70 |
| 2013 | Ryssinge 1 | Tetraploid | 1 | 23.10 | -3.06 |
| 2013 | Ryssinge 1 | Tetraploid | 0 | 24.67 | 2.21 |
| 2013 | Ryssinge 1 | Tetraploid | 0 | 26.00 | 3.17 |
| 2013 | Ryssinge 1 | Tetraploid | 0 | 22.00 | 3.01 |
| 2013 | Ryssinge 1 | Tetraploid | 0 | 20.82 | 2.83 |
| 2013 | Ryssinge 1 | Tetraploid | 0 | 23.33 | 1.03 |
| 2013 | Ryssinge 1 | Tetraploid | 0 | 26.51 | -0.44 |
| 2013 | Ryssinge 1 | Tetraploid | 0 | 27.33 | 1.31 |
| 2013 | Ryssinge 1 | Tetraploid | 0 | 27.65 | 2.66 |
| 2013 | Ryssinge 1 | Tetraploid | 0 | 27.95 | 0.78 |
| 2013 | Ryssinge 1 | Tetraploid | 1 | 20.91 | -0.74 |
| 2013 | Ryssinge 1 | Tetraploid | 1 | 28.15 | -1.61 |
| 2013 | Ryssinge 1 | Tetraploid | 1 | 20.67 | 0.04 |
| 2013 | Ryssinge 1 | Tetraploid | 1 | 20.08 | -3.22 |
| 2013 | Ryssinge 1 | Tetraploid | 1 | 22.00 | 0.71 |
| 2013 | Ryssinge 1 | Tetraploid | 1 | 30.94 | -0.95 |
| 2013 | Ryssinge 1 | Tetraploid | 1 | 20.77 | -0.82 |
| 2013 | Ryssinge 1 | Tetraploid | 0 | 26.12 | -1.10 |
| 2013 | Ryssinge 1 | Tetraploid | 0 | 27.12 | 0.50 |
| 2013 | Ryssinge 3 | Tetraploid | 0 | 20.43 | -1.57 |
| 2013 | Ryssinge 3 | Tetraploid | 0 | 20.62 | 0.12 |
| 2013 | Ryssinge 3 | Tetraploid | 0 | 26.00 | 0.59 |
| 2013 | Ryssinge 3 | Tetraploid | 0 | 21.61 | -1.98 |
| 2013 | Ryssinge 3 | Tetraploid | 0 | 20.45 | -2.97 |
| 2013 | Ryssinge 3 | Tetraploid | 0 | 20.91 | -1.53 |
| 2013 | Ryssinge 3 | Tetraploid | 0 | 24.25 | -2.64 |
| 2013 | Ryssinge 3 | Tetraploid | 0 | 25.76 | -1.51 |
| 2013 | Ryssinge 3 | Tetraploid | 1 | 21.57 | -2.92 |
| 2013 | Ryssinge 3 | Tetraploid | 1 | 25.85 | -3.75 |
| 2013 | Ryssinge 3 | Tetraploid | 0 | 24.67 | 0.90 |
| 2013 | Ryssinge 3 | Tetraploid | 0 | 20.42 | 0.01 |
| 2013 | Ryssinge 3 | Tetraploid | 0 | 20.94 | 0.37 |
| 2013 | Ryssinge 3 | Tetraploid | 0 | 26.00 | -2.78 |
| 2013 | Ryssinge 3 | Tetraploid | 0 | 20.83 | -0.46 |
| 2013 | Ryssinge 3 | Tetraploid | 0 | 20.16 | -2.64 |
| 2013 | Ryssinge 3 | Tetraploid | 0 | 25.07 | -0.80 |
| 2013 | Ryssinge 3 | Tetraploid | 0 | 28.00 | 1.92 |
| 2013 | Ryssinge 3 | Tetraploid | 0 | 29.11 | 1.77 |
| 2013 | Ryssinge 3 | Tetraploid | 0 | 28.80 | 0.69 |
| 2013 | Ryssinge 3 | Tetraploid | 0 | 21.44 | -1.08 |
| 2013 | Ryssinge 3 | Tetraploid | 1 | 25.52 | -0.14 |
| 2013 | Ryssinge 3 | Tetraploid | 0 | 21.20 | -0.74 |
| 2013 | Ryssinge 3 | Tetraploid | 0 | 20.62 | 0.06 |
| 2013 | Ryssinge 3 | Tetraploid | 0 | 29.09 | 0.30 |
| 2013 | Ryssinge 3 | Tetraploid | 0 | 22.00 | -0.64 |
| 2013 | Ryssinge 3 | Tetraploid | 0 | 20.62 | -0.31 |
| 2013 | Ryssinge 3 | Tetraploid | 1 | 22.00 | 0.15 |
